# Novel Coumarin–Nucleobase Hybrids with Potential Anticancer Activity: Synthesis, In Vitro Cell-Based Evaluation, and Molecular Docking

**DOI:** 10.3390/ph17070956

**Published:** 2024-07-17

**Authors:** Maiara Correa de Moraes, Rafaele Frassini, Mariana Roesch-Ely, Favero Reisdorfer de Paula, Thiago Barcellos

**Affiliations:** 1 Laboratório de Biotecnologia de Produtos Naturais e Sintéticos, Universidade de Caxias do Sul, Francisco Getúlio Vargas St., 1130, Caxias do Sul 95070-560, RS, Brazil; maiara.moraes@caxias.ifrs.edu.br; 2 Instituto Federal de Educação, Ciência e Tecnologia do Rio Grande do Sul—Campus Caxias do Sul, Avelino Antônio de Souza, 1730, Caxias do Sul 95043-700, RS, Brazil; 3 Laboratório de Genômica, Proteômica e Reparo de DNA, Universidade de Caxias do Sul, Francisco Getúlio Vargas St., 1130, Caxias do Sul 95070-560, RS, Brazil; rfrassin@ucs.br (R.F.); mrely@ucs.br (M.R.-E.); 4 Laboratório de Desenvolvimento e Controle de Qualidade em Medicamentos, Universidade Federal do Pampa, Campus Uruguaiana, BR 472, Km 592, Uruguaiana 97508-000, RS, Brazil; faveropaula@unipampa.edu.br

**Keywords:** molecular hybridization, coumarin, nucleobases, anticancer, molecular docking

## Abstract

A new series of compounds planned by molecular hybridization of the nucleobases uracil and thymine, or the xanthine theobromine, with coumarins, and linked through 1,2,3-triazole heterocycles were evaluated for their in vitro anticancer activity against the human tumor cell lines: colon carcinoma (HCT116), laryngeal tumor cells (Hep-2), and lung carcinoma cells (A549). The hybrid compound **9a** exhibited better activity in the series, showing an IC50 of 24.19 ± 1.39 μM against the HCT116 cells, with a selectivity index (SI) of 6, when compared to the cytotoxicity against the non-tumor cell line HaCat. The in silico search for pharmacological targets was achieved through molecular docking studies on all active compounds, which suggested that the synthesized compounds possess a high affinity to the Topoisomerase 1–DNA complex, supporting their antitumor activity. The in silico toxicity prediction studies suggest that the compounds present a low risk of causing theoretical mutagenic and tumorigenic effects. These findings indicate that molecular hybridization from natural derivative molecules is an interesting approach to seek new antitumor candidates.

## 1. Introduction

One of the key challenges in medicinal chemistry is the development of more effective, selective. and safer compounds for treating known and new pathologies [1]. Even though there are almost infinite possibilities of novel chemical structures wanted for their bioactive properties’ evaluation, the molecular hybridization of known bioactive entities can be used as an alternative approach to optimize the process. Molecular hybridization involves the rational design and association of two or more pharmacophores into a single molecule, aiming at new molecular entities that allow multiple biological activities, modified selectivity profiles, different or dual modes of action, and reduced undesired side effects [2,3,4,5,6,7,8].

Although molecular hybridization aims to combine the pharmacophoric properties of the distinct “parent” molecules as multi-targeted agents, it is relevant to consider that the new molecular structure of the hybrid differs from the molecular structure of the “parent” molecules. Thus, the comparison of using the combined “parent” pharmacophores as a drug cocktail administration is limited. Such disadvantages include the difficulty of establishing the ratio of activities at different targets or obtaining a clear interpretation of the molecular and cellular basis of the hybrid activity. In addition, a more complex synthesis can also be considered a disadvantage along with the inevitable increase in molecular mass and lipophilicity [6,7].

Coumarin and its natural and synthetic derivatives are a remarkable example of a naturally based occurring heterocycle that presents an extensive range of pharmacological activities, including antibacterial [9], antitubercular [10], sedative–hypnotic [11], antioxidant [12], anti-inflammatory [13], anticoagulant [14], antileishmanial [15], and anticancer [16,17,18,19,20].

The *2H*-chromen-2-one core is considered a privileged structure due to its rigid and conjugated structure. The aromatic ring allows a series of hydrophobic, π–π, CH–π, and cation–π interactions, and the two oxygen atoms in the lactone ring can also hydrogen-bond with amino acid residues in different classes of enzymes and receptors [21]. The relevant pharmacological profile of the *H*-chromen-2-one nucleus is illustrated by its presence in the main backbone of approved drugs such as warfarin (anticoagulant), carbochromen (vasodilator), and novobiocin (antibiotic) [22]. Regarding the molecular hybridization strategy, coumarins have also been employed along with diverse bioactive compounds [4,22,23,24].

Pyrimidine and its derivatives are also a significant and widespread class of nitrogen-containing heterocycles that are an integral part of DNA and RNA building blocks. They play an essential role in the biological process and, consequently, have considerable chemical and pharmacological importance. 5-fluorouracil (5-FU) is an example of a pyrimidine derivative that has been used in cancer treatment. 5-FU, such as other pyrimidine derivatives, acts as an antagonist in the biosynthetic pathways of pyrimidine nucleobases [25], competing for the same binding sites of naturally occurring compounds [26].

1,2,3-Triazoles have been frequently used as an attractive binding unit (linker) between the pharmacophoric units [5]. The 1,2,3-triazole moiety is easily achieved through the dipolar cycloaddition between azides and alkynes [27,28] and also presents several biological activities, which include antibacterial [29], antifungal [30], and anticancer [31]. It is also metabolically stable and capable of forming hydrogen bonds, which could be favorable in binding biomolecular targets [9].

**Figure 1:**
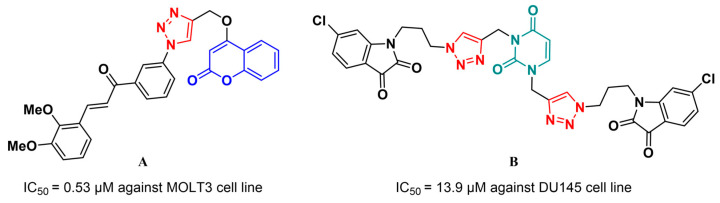
Representative examples of biologically active coumarin (**A**) and uracil-containing compounds (**B**) hybridized through the 1,2,3-triazole heterocycle.

Coumarin and uracil derivatives conjugated to 1,2,3-triazoles have been reported in the literature and evaluated as possible anticancer agents. For instance, coumarin–chalcone hybrids linked by the 1,2,3-triazole ring were synthesized and evaluated as anticancer and antimalarial agents [32]. Example A (Figure 1) showed antiproliferative activity against human acute T lymphoblastic leukemia cell lines (MOLT-3) with an IC_50_ of 0.53 µM. 

An example of a pyrimidine derivative employed in the molecular hybridization strategy is the uracil–isatin conjugates hybridized via the 1,2,3-triazole bridge [33]. Among the series prepared in this study, compound B (Figure 1) showed considerable selectivity for the human prostate cancer cell line (DU145) with an IC_50_ of 13.9 µM.

Considering the pharmacological relevance of the coumarin and pyrimidine derivatives, in this work, we explored their molecular hybridization linked by a triazole unit aiming for a non-cleavable conjugated pharmacophore. 

Thus, we investigate the potential anticancer activities of the novel coumarin–nucleobase hybrids against three human cell lines, colon carcinoma (HCT116), laryngeal tumor (Hep-2), and lung carcinoma (A549), and one non-tumor cell line, namely HaCat (human keratinocyte), using the colorimetric MTT assay.

## 2. Results and Discussion

### 2.1. Chemistry

**Figure 2:**
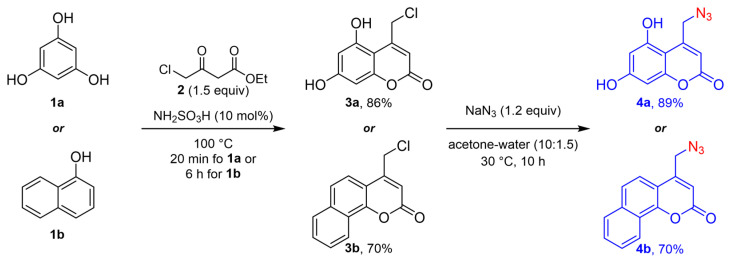
Synthesis of 4-(azidomethyl)coumarins **4a** and **4b**.

The synthetic protocol to obtain the target hybrid compounds started with the Pechmann condensation of the phenols phloroglucinol (**1a**) and 1-naphthol (**1b**) with the β-ketoester ethyl 4-chloroacetoacetate (**2**) to achieve the 4-chloromethylcoumarins **3a** and **3b** with 86 and 70% yield, respectively. This procedure was carried out in the absence of solvent and using sulfamic acid as a catalyst [34]. Then, the azide group was installed by the nucleophilic displacement of the chlorine atom [35], affording the 4-(azidomethyl)coumarins **4a** and **4b** with 89 and 86% yields, respectively, as shown in Figure 2.

Next, we prepare the portions constituted by the *N1*-propargylated uracil (**6a**), *N1*-propargylated thymine (**6b**), or *N1*,*N2*-dipropargylated uracil (**6c**). Initially, we envisioned the monopropargylation reaction of the nucleobases at the *N1* position, retaining the hydrogen at the *N3* position free due to its importance in biological activity. The preservation of the hydrogen at the *N3* position is intended to maintain the same hydrogen-bond pairing scheme presented by uracil. In addition, the propargylation at the *N1* position is necessary to introduce lipophilic groups or to bind other groups to the nucleobase skeleton [26]. 

**Figure 3:**
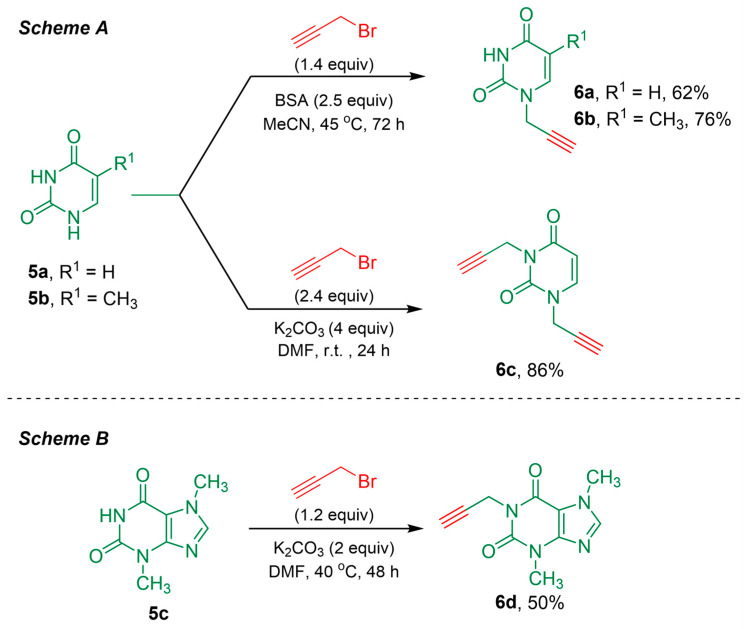
Synthetic methodologies employed to prepare the *N1*-propargylated uracil **6a**, the *N1*-propargylated thymine **6b**, the *N1*,*N2*-dipropargylated uracil **6c**, and the propargylated theobromine **6d**.

Thus, to selectively prepare the *N1*-propargylated nucleobases **6a,b**, the bis(trimethylsilyl)acetamide (BSA) was employed as a base, using acetonitrile as a solvent, at 45 °C for 72 h [36]. This methodology allows us to obtain the desired compounds as unique products with a 62% and 76% yield for **6a** and **6b**, respectively (Figure 3, Scheme A). Afterward, the *N1*,*N2*-dipropargylated uracil **6c** was synthesized in order to obtain the hybrid compounds bis-(coumaronyl-triazolyl)uracil, which is based on similar molecular structures that present anticancer activity [33]. In this case, the dipropargylation reaction was carried out using the simple base K_2_CO_3_ in anhydrous DMF at room temperature for 24 h. The *N1*,*N2*-dipropargylated uracil **6c** was obtained with an 86% yield (Figure 3, Scheme A).

In addition to uracil and thymine, we also decided to investigate the influence of the theobromine (**5c**, 3,7-dimethylxanthine) moiety in the activity. The propargylated theobromine (**6d**) was prepared using K_2_CO_3_ as a base, in anhydrous DMF, at 40 °C [33]. However, a longer reaction time of 48 h was necessary to achieve only 50% yield (Figure 3, Scheme B).

Theobromine is an alkaloid of the family methylxanthines. It is found mainly in cocoa products and has a diuretic action [37]. In addition, theobromine derivatives demonstrated potential antitumor activities through multiple mechanisms and could reverse resistance to multiple drugs. In view of this potential, theobromine derivatives can also lead to potent antitumor agents that are selective and have low toxicity [38,39].

**Figure 4:**
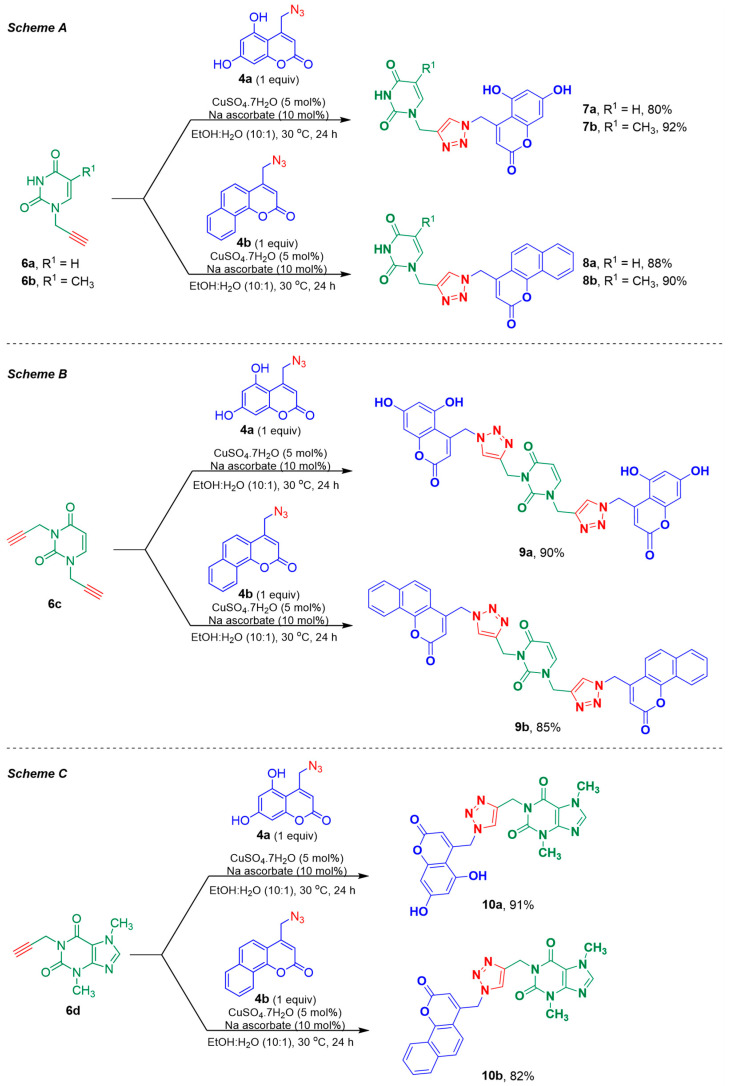
A Cu(I)-catalyzed [3+2]-cycloaddition reaction between the 4-(azidomethyl)coumarins **4a,b**, and the *N1*-propargylated uracil **6a**, *N1*-propargylated thymine **6b** (Scheme A), *N1*,*N2*-dipropargylated uracil **6c** (Scheme B), and the propargylated theobromine **6d** (Scheme C), leading to the corresponding target hybrid molecules.

The propargylated nucleobases **6a–d** and the 4-(azidomethyl)coumarins **4a,b** were hybridized through a 1,2,3-triazole ring by a Cu(I)-catalyzed [3+2]-cycloaddition reaction, as outlined in Figure 4. The click reaction was successfully employed for all intermediates, using the simple copper(II) sulfate and sodium ascorbate system to generate the copper(I) catalyst. All the reactions were carried out in ethanol–water (10:1), at 30 °C, for 24 h.

After the reaction completion, the target compounds were simply purified by pouring the reaction contents into an ice-water mixture. The precipitated products were filtered and presented a high purity degree, as observed by ^1^H NMR. However, when impurities were noticed, they were removed by a short pad of silica gel. The target compounds **7a,b**, **8a,b**, **9a,b**, and **10a,b** were prepared from good to excellent yield, as shown in Figure 4.

All compounds were characterized by ^1^H and ^13^C NMR, HRMS, and melting point. The NMR and HRMS spectra for the final compounds are reported in the Supplementary Materials.

### 2.2. Inclusion Complex

Before the cytotoxic evaluation, it was noticed that the target compounds showed low solubility in the cell medium, even the *N1*-(cumaronyltriazolyl)uracils **7a,b** and **8a,b**, with the free hydrogen at the *N3* position. In order to enhance the solubility, we proposed the use of non-toxic (2-hydroxylpropyl)-β-cyclodextrin (HP-β-CD) as a complexing agent. The HP-β-CD is suitable for cell cultures and has been used to enhance the solubility of non-polar compounds, such as vitamins and hormone derivatives [40]. Hydrophobic molecules are incorporated into the cavity by water molecule displacement, thus favoring the solubility in the medium. During the cytotoxic experiment, the reverse process occurs due to low concentration, releasing the active compound. Thus, the complex compounds were prepared by vigorous mixing of a solution of the targets in ethanol with an equimolar amount of HP-β-CD in water. After 15 h of vigorous mixing, a clean solution was noticed, in which, in turn, the solvent was removed under a vacuum, and the residual water was removed by the lyophilization method to obtain a solid. The complex compounds were characterized by ^1^H NMR, and besides the presence of HP-β-CD signals, no significant changes in the chemical shift of the signals of the target compounds were noticed (Figure S26). 

### 2.3. Biological Activities

#### Cytotoxicity Assay

The potential antitumor activity of eight new hybrids was evaluated through colorimetric microculture MTT assay against three tumor cell lines: human colon carcinoma cells (HCT116), human laryngeal tumor cells (Hep-2), and human lung carcinoma cells (A549). The hybridized compounds were also assayed against the non-tumor cell line HaCat (human keratinocyte) in order to obtain an indication of their selective cytotoxicity. Also, the commercial anticancer drug doxorubicin was employed as a positive drug standard to compare the cytotoxicity activities. Each experiment was carried out in triplicate on three different days. The selectivity index (SI) was calculated as the ratio between the IC_50_ (concentration that reduces cell viability by 50%) of HaCat (non-tumor line) and the IC_50_ of the tumor lines. The results are outlined in Table 1.

The mono *N1*-(coumaronyltriazolyl)uracils **7a,b** and **8a,b** did not show activity against the tumor cell lines, with the exception of compound **8b**, which showed poor cytotoxicity only against HCT116 cells with an IC_50_ of 87.58 ± 1.94 μM. Compound **9a**, resulting from the molecular hybridization of the uracil and two coumarins derived from phloroglucinol, exhibits the highest cytotoxicity against HCT116 cells with an IC_50_ of 24.19 ± 1.39 μM for a 48 h experiment (Table 1, entry 1). Interestingly, compound **9a** did not exhibit appreciable cytotoxicity against the non-tumor cells HaCat, showing a good selectivity index (SI) of 6.0. No cytotoxicity effect was observed for compound **9a** against A549 cells in 24 and 48 h treatments. For Hep-2 cells, an IC_50_ of 51.55 ± 1.71 μM was noticed for 24 h of exposure.

Compound **9b**, which is an analog to **9a**, but with the coumarin moiety derived from 1-naphthol, presented an IC_50_ for HCT116 cells of 54.50 ± 1.74 and 59.17 ± 1.77 μM for 24 and 48 h treatments. It was the only compound in the series that showed activity against A549 cells, with an IC_50_ of 51.03 ± 1.71 μM for 24 h of exposure. Besides the moderated cytotoxicity, the selectivity index when compared to HaCat cells was 63. No appreciative cytotoxicity was observed for Hep-2 cells.

Compounds **10a,b**, both derived from the nucleobase theobromine, showed activity against HCT116 cells. However, compound **10b** showed higher potency with an IC_50_ 40.98 ± 1.61 μM for 24 h of exposure with a selectivity index of 6. Compounds with SI ≥ 10 can be considered selective [41]. Compound **10a** showed an IC_50_ of 78.26 ± 1.89 in 24 h of exposure for HCT116 cells, and an IC_50_ of 56.55 ± 1.75 μM for Hep-2 cells, with a selectivity index of 4 and 5, respectively. None of the tested compounds showed cytotoxicity against the non-tumor cell line (HaCat), even at the highest evaluated concentration of 100 μM at all exposure times tested. Also, the reference compound doxorubicin was used as a positive drug standard to compare the cytotoxicity activities. Besides being used as an effective antitumor agent, doxorubicin exhibits well-documented toxic effects, including tissue ulceration, necrosis, and cardiac toxicity [42]. For the HCT116 cell line, compound **9a** showed a moderated activity (approximately 20-fold) when compared to doxorubicin (IC_50_ of 1.23 ± 0.05 μM).

### 2.4. In Silico Studies

The Osiris Property Explorer^®^ (Idorsia Pharmaceuticals Ltd., Allschwil, Switzerland) [43] software was used to calculate the mutagenic, tumorigenic, irritant, and toxicant reproductive system effects of compounds **9a,b** and **10a,b**, and therefore, to predict the toxicity risks. The calculations are based on the functional group similarity between the query molecules within the compounds present in the software database. The results were compared with the standard H_2_O_2_ and are outlined in Table 2. 

The evaluation showed that compounds **9a** and **9b** showed a low risk of causing theoretical mutagenic and also tumorigenic effects. No risk of irritant effect was determined. Only compound **9a** showed a medium risk of causing an effect on the reproductive system. All compounds showed lower predicted toxic effects in comparison with H_2_O_2_. These results suggest that the compounds present a low potential to cause theoretical toxicity risks and, thus, can be submitted to drug design development.

In order to understand the cytotoxicity presented by the compounds in the MTT assay, we decided to employ computational techniques to suggest the most likely biological target. The chemical similarity ensemble algorithm (SEA) [44] was employed based on the hypothesis of the identification of candidate target proteins to interact with the shared scaffolds of uracil, coumarin, and triazole, such as (1-((1-((5,7-dihydroxy-2-oxo-2H-chromen-4-yl)methyl)-1H-1,2,3-triazol-4-yl)methyl)-3-methylpyrimidine-2,4(1H,3H)-dione), from the structures of **9a**, **9b**, **10a**, and **10b**. The results indicated the potential of several protein targets, in which the main target model is related to the Topoisomerase 1–DNA complex (Topo 1–DNA complex), which showed a *p*-value of 3.83 × 10^−31^ and a maximum Tanimoto coefficient (max-TC) of 0.29. All predicted results are shown in Table 3.

Topoisomerase inhibitors have been shown to be potent antineoplastic agents. The explanation for this potential is due to the mechanism of action of topoisomerase-targeted drugs; the higher the cellular concentration of topoisomerases, the more lethal these drugs become. Tumor cells grow faster and generally express higher concentrations of topoisomerase than normal cells. Therefore, drugs generate more DNA breaks and are more toxic to tumor cells [45].

Compounds containing triazole, coumarin, or uracil nuclei in their structures have shown antitumor activity attributed to topoisomerase inhibitory activity [46,47,48], which could indicate the importance of these cores in the construction of new molecules aimed at this biological target. Thus, according to the obtained results, the Topo 1-DNA complex was selected as the target protein in further docking studies.

### 2.5. Molecular Modeling and Docking Studies

For the docking analyses, the lowest-energy molecular conformers for the most active compounds **9a,b** and **10a,b** were generated by Spartan’08 modeling software (version 1.2.0, Wavefunction Inc., Irvine, CA, USA). Further, the docking studies were performed to identify the individual pose that presents lower energy that selectively binds to the Topo 1-DNA complex active site. The molecular docking analysis was accomplished by using the iGEMDOCK software (version 2.1) [49] and applying the generic evolutionary method (GA). The empirical scoring function was measured using the total sum of the energies of van der Waal forces (VDW), hydrogen bond (H-bond), and electrostatic interactions occurring between the compounds and the target protein, as shown in Table 4.

Compound **9a** complexed with the Topo 1-DNA complex was the ligand with the lowest energy in the series, with a total binding energy of −118.5 kcal mol^−1^. Ligands **9b** and **10a** show similar total binding energy values, which are −113.3 and −113.7 kcal mol^–1^, respectively. However, for ligand **9b**, the contribution of van der Waal energy was larger than that of ligand **10a**. In turn, the contribution of H-bond energy for ligand **10a** was larger than for ligand **9b**. Ligand **10b** showed the lowest total binding energy in the series, with a value of −102.7 kcal mol^−1^.

**Figure 5:**
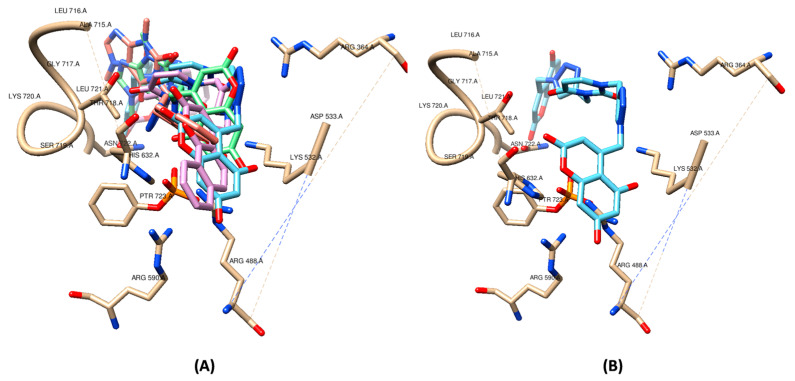
Predicted binding of most active in vitro compounds **9a,b** and **10a,b** poses (**A**) and **9a** pose (**B**) in active site of Topo 1-DNA complex (PDB code: 1K4S). Graphic visualization obtained using UCSF Chimera (v.1.10.1) [51].

Considering the interaction with the amino acid residues in the active site of the Topo 1-DNA complex, the docking studies reveal that the main interactions occur with the residues ARG 364, ARG 488, LYS 532, ASP 533, ILE 535, HIS 632, THR 718, LEU 721, ASN 722, TYR 723 (O-phospho-L-tyrosine), and LYS 571, as illustrated in Figure 5A. The measured energies are represented in Table 5. The residues ARG 364, LYS 532, ASP 533, ASN 722, and TYR 723 are the same as those observed in docking studies performed by Laco (2011) [50], who suggest the same active site of the Topo 1-DNA complex herein studied. 

In the post-screening analysis using Residues Consensus Analysis, HIS 632 was detected as the main residue evolved in this ligand-receptor binding (with Z-score 1.94 and WPharma 1.00).

Van der Waals interactions are the main type between the ligands and the residues in the binding site of the Topo 1-DNA complex. Some relevant interactions to compound poses are observed in LYS 532, HIS 632, and LEU 721. The main molecular regions of interaction between **9a** and the Topo 1-DNA complex in the in silico model are shown in Figure 5B. The carbonyl group from the coumarin moiety showed relevant interaction with HIS 632, while the uracil moiety interacted with ARG 364 and THR 718. One of the triazole rings showed interaction with ARG 364 from the Topo 1-DNA complex. Thus, based on the results from the docking studies, the most active compounds are potential ligands and fit as candidates as Topo 1-DNA complex inhibitors.

## 3. Materials and Methods

### 3.1. Materials and Instrumentation

All reagents and solvents used were purchased from commercial suppliers (Sigma-Aldrich^®^, São Paulo, Brazil) and used without further purification. The NMR experiments were performed on a Fourier 300 FT-NMR spectrometer (Bruker Daltonics, Bremen, German; 7.05 Tesla, 300 MHz for the ^1^H nucleus and 75 MHz for the ^13^C nucleus). The chemical shifts (δ) values are expressed in part per million (ppm) and are relative to DMSO-d6 (2.50 ppm for ^1^H NMR spectra and 39.52 ppm for ^13^C NMR spectra). The coupling constants are reported in Hz. The spectra were acquired at a temperature of 293 K, using 5 mm quartz tubes. For the NMR data acquisition and processing, the TopSpin™ software (version 4.0.7 Bruker BioSpin, Rheinstetten, Germany) was used. The high-resolution electrospray ionization mass spectrometry (ESI-QTOF) analyses were performed on a micrOTOF-Q II instrument (Bruker Daltonics, Billerica, MA, USA) in a positive mode under the following conditions: capillary and cone voltages were set to +3500 V and +40 V, respectively, with a de-solvation temperature of 200 °C. The samples were solubilized in HPLC-grade methanol containing 0.1% formic acid and injected into the ESI source by means of a syringe pump at a flow rate of 5.0 µL min 1. Melting points were recorded on a capillary melting point apparatus (Fisatom, model 431, São Paulo, Brazil), with a measurement range from 50 °C to 350 °C, and they are uncorrected. 

### 3.2. The General Procedure for the Preparation of the 4-(Chloromethyl)-coumarins ***3a,b***

Phenol (**1a** or **1b**) (5 mmol), ethyl 4-chloroacetoacetate (7.5 mmol), and sulfamic acid (10 mol%, 0.0485 g) were added to a 30 mL glass vial and tightly sealed with a Teflon cap. The reaction mixture was heated at 100 °C, for 20 min (**1a**) or 6 h (**1b**). After, the reaction mixture was cooled to room temperature and dissolved in 25 mL of hot ethanol, filtered, and poured into an ice-water mixture (100 mL). The precipitate that formed was filtered and recrystallized from ethanol.

#### 3.2.1. 4-(Chloromethyl)-5,7-dihydroxy-2*H*-chromen-2-one (**3a**)

White solid (970 mg, 86%); mp 231-233 °C (lit. [34], 240–242 °C); ^1^H NMR (300 MHz, acetone-d_6_) *δ* 5.06 (d, *J* 1.2 Hz, 2H), 6.27 (t, *J* 1.2 Hz, 1H), 6.32 (d, *J* 2.4 Hz, 1H), 6.38 (d, *J* 2.4 Hz, 1H), 9.34 (s, 1H, OH), 9.85 (s, 1H, OH); ^13^C NMR (75 MHz, DMSO-d_6_) *δ* 45.10, 94.89, 99.32, 99.88, 108.85, 152.14, 156.59, 157.25, 160.19, 161.63; HRMS (ESI+) *m*/*z* calcd. for C_10_H_8_ClO_4_ [M+H]^+^: 227.0105, found: 227.0101.

#### 3.2.2. 4-(Chloromethyl)-2*H*-benzo[*h*]chromen-2-one (**3b**)

Light brown solid (850 mg, 70%); mp 154–156 °C (lit. [34] 159-161 °C); ^1^H NMR (300 MHz, DMSO-d_6_) *δ* 5.12 (s, 2H, CH_2_), 6.79 (s, 1H, CH), 7.76–7.69 (m, 2H), 7.91–7.83 (m, 2H), 8.06–8.03 (m, 1H), 8.37–8.34 (m, 1H); ^13^C NMR (75 MHz, DMSO-d_6_) *δ* 41.66, 112.78, 114.85, 120.89, 121.61, 122.23, 124.07, 127.53, 127.98, 128.97, 134.36, 150.32, 151.44, 159.52; HRMS (ESI+) *m*/*z* calcd. for C_14_H_10_ClO_2_ [M+H]^+^: 245.0363, found: 245.0369.

### 3.3. The General Procedure for the Preparation of 4-(Azidomethyl)-coumarins (***4a,b***)

In a 50 mL round-bottomed flask equipped with a magnetic stirrer, 4-(chloromethyl)-coumarins **3a,b** (2 mmol) were taken in 4 mL of acetone. After, a solution of sodium azide (2.4 mmol) in 0.6 mL of water was added dropwise with a continuous stirring, which was kept for an additional 10 h at 30 °C. The reaction mixture was poured into an ice-water mixture. The precipitate that formed was filtered and recrystallized from ethanol [35].

#### 3.3.1. 4-(Azidomethyl)-5,7-dihydroxy-2*H*-chromen-2-one (**4a**)

White solid (414 mg, 89%); mp 215–217 °C (lit. [52] 220–221 °C); ^1^H NMR (300 MHz, DMSO-d_6_) *δ* 4.87 (d, *J* 1.1 Hz, 2H, CH_2_), 6.05 (t, *J* 1.1 Hz, 1H, CH), 6.21 (d, *J* 2.4 Hz, 1H), 6.27 (d, *J* 2.4 Hz, 1H), 10.42 (s, 1H, OH), 10.88 (s, 1H, OH); ^13^C NMR (75 MHz, DMSO-d_6_) *δ* 52.79, 94.82, 99.11, 100.07, 106.76, 151.76, 156.52, 157.31, 160.09, 161.56; HRMS (ESI+) *m*/*z* calcd. for C_10_H_7_N_3_O_4_Na [M+Na]^+^: 256.0328, found: 256.0325.

#### 3.3.2. 4-(Azidomethyl)-2*H*-benzo[*h*]chromen-2-one (**4b**)

Light brown solid (431 mg, 86%); mp 136–138 °C (lit. [53] 131 °C); ^1^H NMR (300 MHz, DMSO-d_6_) *δ* 4.97 (s, 2H, CH_2_), 6.63 (s, 1H, CH), 7.76-7.70 (m, 3H), 7.88 (d, *J* 8.4 Hz 1H), 8.06–8.04 (m, 1H), 8.38-8.35 (m, 1H); ^13^C NMR (75 MHz, DMSO-d_6_) *δ* 49.97, 112.81, 112.92, 120.45, 121.59, 122.15, 124.14, 127.51, 127.97, 128.90, 134.33, 150.05, 150.79, 159.44; HRMS (ESI+) *m*/*z* calcd. for C_14_H_10_N_3_O_2_ [M+H]^+^: 252.0767, found: 252.0762.

### 3.4. General Procedure for Monopropargylation of Uracil and Thymine and Synthesis of ***6a*** and ***6b***

In a 50 mL round-bottomed flask, equipped with a magnetic stirrer, uracil (**5a**, 0.560 g, 5 mmol) or thymine (**5b**, 0.631 g, 5 mmol) was suspended in dry acetonitrile (15 mL), *N*,*O*-bis-(trimethylsilyl)acetamide (BSA, 3.06 mL, 12.5 mmol) was added, and the mixture was stirred until a clear solution was obtained. Subsequently, propargyl bromide (80 wt.% in toluene, 0.615 mL, 6.9 mmol) was added dropwise and the reaction mixture was heated at 45 °C for 72 h. The acetonitrile was evaporated under a vacuum, and the residue was treated with a saturated aqueous NH_4_Cl solution (15 mL) and extracted with CH_2_Cl_2_. The organic phase was dried with anhydrous Na_2_SO_4_ and concentrated under a vacuum. The crude product was purified by recrystallization from CH_2_Cl_2_/hexane (1:2 *v*/*v*).

#### 3.4.1. 1-(Prop-2-yn-1-yl)pyrimidine-2,4(1*H*,3*H*)-dione (**6a**)

White solid (465 mg, 62%); mp 153–155 °C (lit. [36] 169-170 °C); ^1^H NMR (300 MHz, DMSO-d_6_) *δ* 3.42 (t, *J* 2.4 Hz, 1H), 4.50 (d, *J* 2.4 Hz, 2H), 5.61 (d, *J* 7.8 Hz 1H), 7.69 (d, *J* 7.8 Hz 1H), 11.38 (br, 1H, NH); ^13^C NMR (75 MHz, DMSO-d_6_) *δ* 36.67, 75.89, 78.51, 101.71, 144.57, 150.43, 163.62; HRMS (ESI+) *m*/*z* calcd. for C_7_H_7_N_2_O_2_ [M+H]^+^: 151.0502, found: 151.0507.

#### 3.4.2. 5-Methyl-1-(prop-2-yn-1-yl)pyrimidine-2,4(1*H*,3*H*)-dione (**6b**)

White solid (620 mg, 76%); mp 150–152 °C (lit. [36] 155–157 °C); ^1^H NMR (300 MHz, DMSO-d_6_) *δ* 1.76 (d, *J* 1.0 Hz, 3H), 3.40 (t, *J* 2.4 Hz, 1H), 4.46 (d, *J* 2.4 Hz, 2H), 7.56 (d, *J* 1.0 Hz, 1H), 11.38 (br, 1H, NH); ^13^C NMR (75 MHz, DMSO-d_6_) *δ* 11.98, 36.36, 75.69, 78.70, 109.43, 140.19, 150.40, 164.19; HRMS (ESI+) *m*/*z* calcd. for C_8_H_9_N_2_O_2_ [M+H]^+^: 165.0658, found: 165.0654.

### 3.5. Synthesis of 1,3-Di(prop-2-yn-1-yl)pyrimidine-2,4(1H,3H)-dione (***6c***)

In a 50 mL round-bottomed flask, equipped with a magnetic stirrer, a suspension of potassium carbonate (4 mmol, 0.552 g) in anhydrous DMF (10 mL) and uracil (1 mmol, 0.112 g) was added. The mixture was stirred for 1 h at room temperature. Afterward, propargyl bromide (80 wt.% in toluene, 0.212 mL, 2.4 mmol) was added, and the reaction mixture was kept under stirring for 24 h at room temperature. After the reaction completion, the mixture was treated with brine (20 mL) and extracted with ethyl acetate (3 × 20 mL). The organic phase was dried with anhydrous Na_2_SO_4_ and concentrated under reduced pressure. The product 6c was purified by chromatography column using hexane/ethyl acetate (65:35) and obtained as a white solid (161 mg, 86%); mp 98–100 °C (lit. [54] 102–104 °C); ^1^H NMR (300 MHz, CDCl_3_) *δ* 2.19 (t, *J* 2.4 Hz, 1H), 2.52 (t, *J* 2.4 Hz, 1H), 4.61 (d, *J* 2.4 Hz, 2H), 4.72 (d, *J* 2.4 Hz, 2H), 5.85 (d, *J* 7.8 Hz, 1H), 7.47 (d, *J* 7.8 Hz 1H); ^13^C NMR (75 MHz, acetone-d_6_) *δ* 30.51, 38.38, 71.75, 75.53, 78.19, 79.45, 101.95, 143.33, 151.00, 162.09; HRMS (ESI+) *m*/*z* calcd. for C_10_H_9_N_2_O_2_ [M+H]^+^: 189.0658, found: 189.0668.

### 3.6. Synthesis of 3,7-Dimethyl-1-propargylxanthine (***6d***)

In a 100 mL round-bottomed flask, equipped with a magnetic stirrer, a suspension of potassium carbonate (29.70 mmol, 4.10 g) in anhydrous DMF (30 mL) and theobromine (14.85 mmol, 2.67g) was added. The mixture was stirred for 1 h at room temperature followed by the addition of propargyl bromide (80 wt.% in toluene, 1.58 mL, 17.82 mmol). The reaction mixture was heated at 40 °C and stirred for 48 h. After the reaction completion, the mixture was treated with brine (30 mL) and extracted with ethyl acetate (4 × 20 mL). The organic phase was dried with anhydrous Na_2_SO_4_ and concentrated under reduced pressure. The product 6d was purified by recrystallization from hexane/ethyl acetate (1:1 *v*/*v*), and obtained as a white solid (1.61 g, 50%); mp 204–205 °C (lit. [55] 209 °C); ^1^H NMR (300 MHz, DMSO-d_6_) *δ* 3.09 (t, *J* 2.4 Hz, 1H), 3.42 (s, 3H), 3.88 (s, 3H), 4.58 (d, *J* 2.4 Hz, 2H), 8.05 (s, 1H); ^13^C NMR (75 MHz, DMSO-d_6_) *δ* 29.93, 30.36, 33.70, 73.26, 80.03, 106.89, 143.77, 148.89, 150.64, 153.88; HRMS (ESI+) *m*/*z* calcd. For C_10_H_10_N_4_O_2_Na [M+Na]^+^: 241.0695, found: 241.0690.

### 3.7. General Procedure for Preparation of 1-(Coumaronyl-triazolyl)uracil ***7a,b***, 1-(Coumaronyl-triazolyl)thymine ***8a,b***, and 1-(Coumaronyl-triazolyl)theobromine ***10a,b***

To a stirred solution of **6a**, **6b**, or **6d** (1 mmol) and 4-(azidomethyl)-coumarins **4a** or **4b** (1 mmol, 0.233 g for **4a** or 0.251 g for 4b) in ethanol–water (10:1, 10 mL), copper sulfate (0.027 mmol, 0.0068 g) and sodium ascorbate (0.072 mmol, 0.0125 g) were added. The reaction mixture was kept under stirring at 30 °C for 24 h. After reaction completion, as indicated by TLC, the mixture was poured into ice water, and the formed precipitate was filtered and freeze-dried under a vacuum.

#### 3.7.1. 1-((1-((5,7-Dihydroxy-2-oxo-2*H*-chromen-4-yl)methyl)-1*H*-1,2,3-triazol-4-yl)methyl) Pyrimidine-2,4(1*H*,3*H*)-dione (**7a**)

White solid (306 mg, 80%); mp 230 °C with degradation; ^1^H NMR (300 MHz, DMSO-d_6_) *δ* 4.64 (s, 1H), 4.99 (s, 2H), 5.59 (d, *J* 7.7 Hz, 1H), 5.99 (s, 1H), 6.03 (s, 2H), 6.16 (s, 1H), 7.77 (d, *J* 7.7 Hz, 1H), 8.21 (s, 1H); ^13^C NMR (75 MHz, DMSO-d_6_) *δ* 42.6, 52.3, 62.8, 92.9, 99.8, 100.5, 101.3, 103.1, 125.0, 142.8, 145.7, 150.8, 153.7, 156.7, 160.5, 162.9, 163.8; HRMS (ESI+) *m*/*z* calcd. for C_17_H_13_N_5_O_6_Na [M+Na]^+^: 406.0758, found: 406.0751.

#### 3.7.2. 1-((1-((5,7-Dihydroxy-2-oxo-2*H*-chromen-4-yl)methyl)-1*H*-1,2,3-triazol-4-yl)methyl)-5-methylpyrimidine-2,4(1*H*,3*H*)-dione (**7b**)

White solid (365 mg, 92%); mp 285 °C with degradation; ^1^H NMR (300 MHz, DMSO-d_6_) *δ* 1.76 (s, 3H), 4.80 (s, 1H), 4.95 (s, 2H), 5.94 (s, 2H), 6.22 (d, *J* 2.0 Hz, 1H), 6.30 (d, *J* 2.0 Hz, 1H), 7.65 (s, 1H), 8.19 (s, 1H), 10.56 (bs, 1H, OH), 11.07 (bs, 1H, OH), 11.32 (s, 1H, NH); ^13^C NMR (75 MHz, DMSO-d_6_) *δ* 12.1, 42.5, 52.2, 94.9, 99.3, 100.1, 105.9, 109.1, 125.1, 141.4, 143.0, 150.9, 152.6, 156.5, 157.7, 160.1, 162.0, 164.5; HRMS (ESI+) *m*/*z* calcd. for C_18_H_16_N_5_O_6_ [M+H]^+^: 398.1095, found: 398.1090.

#### 3.7.3. 1-((1-((2-Oxo-2*H*-benzo[*h*]chromen-4-yl)methyl)-1*H*-1,2,3-triazol-4-yl)methyl) Pyrimidine-2,4(1*H*,3*H*)-dione (**8a**)

White solid (352 mg, 88%); mp 235 °C with degradation; ^1^H NMR (300 MHz, DMSO-d_6_) *δ* 4.98 (s, 2H), 5.59 (dd, *J* 7.8 Hz and *J* 2.2 Hz, 1H), 5.94 (s, 1H), 6.07 (s, 2H), 7.71–7.92 (m, 5H), 8.04–8.07 (m, 1H), 8.29 (s, 1H), 8.36-8.39 (m, 1H), 11.34 (s, 1H, NH); ^13^C NMR (75 MHz, DMSO-d_6_) *δ* 42.6, 49.6, 101.3, 112.7, 113.2, 120.3, 121.7, 122.1, 124.3, 124.8, 127.6, 128.0, 129.1, 134.4, 143.2, 145.6, 150.1, 150.8, 150.9, 159.3, 163.7; HRMS (ESI+) *m*/*z* calcd. for C_21_H_16_N_5_O_4_ [M+H]^+^: 402.1197, found: 402.1199.

#### 3.7.4. 1-((1-((2-Oxo-2*H*-benzo[*h*]chromen-4-yl)methyl)-1*H*-1,2,3-triazol-4-yl)methyl)-5-methylpyrimidine-2,4(1*H*,3*H*)-dione (**8b**)

White solid (373 mg, 90%); mp 270–272 °C; ^1^H NMR (300 MHz, DMSO-d_6_) *δ* 1.74 (s, 3H), 4.94 (s, 2H), 5.94 (s, 1H), 6.06 (s, 2H), 7.65 (s, 1H), 7.72-7.75 (m, 2H), 7.83 (d, *J* 8.9 Hz, 1H), 7.90 (d, *J* 8.9 Hz, 1H), 8.04–8.07 (m, 1H), 8.28 (s, 1H), 8.35-8.38 (m, 1H), 11.33 (s, 1H, NH); ^13^C NMR (75 MHz, DMSO-d_6_) *δ* 12.0, 42.4, 49.6, 108.9, 112.7, 113.3, 120.3, 121.6, 122.1, 124.2, 124.8, 127.6, 128.0, 129.1, 134.4, 141.2, 143.3, 150.1, 150.8, 150.9, 159.3, 164.3; HRMS (ESI+) *m*/*z* calcd. for C_22_H_18_N_5_O_4_ [M+H]^+^: 416.1353, found: 416.1350.

#### 3.7.5. 1-((1-((5,7-Dihydroxy-2-oxo-2*H*-chromen-4-yl)methyl)-1*H*-1,2,3-triazol-4-yl)methyl)-3,7-dimethyl-3,7-dihydro-1*H*-purine-2,6-dione (**10a**)

White solid (410 mg, 91%); mp 315 °C with degradation; ^1^H NMR (300 MHz, DMSO-d6) δ 3.42 (s, 3H), 3.88 (s, 3H), 4.77 (s, 1H), 5.16 (s, 2H), 5.60 (s, 2H), 6.21 (d, J 2.2 Hz, 1H), 6.29 (d, J 2.2 Hz, 1H), 8.04 (s, 1H), 8.10 (s, 1H), 10.50 (bs, 1H, OH), 11.05 (bs, 1H, OH); ^13^C NMR (75 MHz, DMSO-d6) δ 29.5, 33.2, 36.0, 52.0, 94.8, 99.1, 99.9, 105.7, 106.7, 124.8, 143.1, 143.7, 148.4, 150.7, 152.6, 154.1, 156.3, 157.5, 159.9, 161.8; HRMS (ESI+) *m*/*z* calcd. for C_20_H_18_N_7_O_6_ [M+H]^+^: 452.1313, found: 452.1320.

#### 3.7.6. 3,7-Dimethyl-1-((1-((2-oxo-2*H*-benzo[*h*]chromen-4-yl)methyl)-1*H*-1,2,3-triazol-4-yl)methyl)-3,7-dihydro-1*H*-purine-2,6-dione (**10b**)

Pale yellow solid (384 mg, 82%); mp 288–290 °C; ^1^H NMR (300 MHz, DMSO-d6) δ 3.42 (s, 3H), 3.83 (s, 3H), 5.15 (s, 2H), 5.93 (s, 1H), 6.03 (s, 2H), 7.70-7.78 (m, 2H), 7.85 (d, *J* 8.8 Hz, 1H), 7.901(d, *J* 8.8 Hz, 1H), 8.04–8.08 (m, 2H), 8.19 (s, 1H), 8.37–8.40 (m, 1H); ^13^C NMR (75 MHz, DMSO-d6) δ 29.5, 33.2, 35.9, 49.5, 106.6, 112.8, 113.3, 120.4, 121.7, 122.1, 124.2, 124.6, 127.6, 128.0, 129.1, 134.4, 143.1, 143.9, 148.4, 150.1, 150.7, 151.0, 154.1, 159.4; HRMS (ESI+) *m*/*z* calcd. for C_24_H_20_N_7_O_4_ [M+H]^+^: 470.1571, found: 470.1566.

### 3.8. General Procedure for the Preparation of 1,3-Bis-(coumaronyl-triazolyl)uracil ***9a*** and ***9b***

To a stirred solution of 1,3-di(prop-2-yn-1-yl)pyrimidine-2,4(1*H*,3*H*)-dione **6c** (1 mmol, 0.188 g) and 4-(azidomethyl)-coumarin **4a** or **4b** (2 mmol, 0.466 g for **4a**; 0.502 g for **4b**) in ethanol–water (10:1, 10 mL), CuSO_4_.7H_2_O (0.055 mmol, 0.0136 g) and sodium ascorbate (0.143 mmol, 0.025 g) were added. The reaction mixture was kept under stirring at 30 °C for 24 h. After reaction completion, as indicated by TLC, the mixture was poured into ice water, and the resulting precipitate was filtered and freeze-dried under a vacuum.

#### 3.8.1. 1,3-Bis((1-((5,7-dihydroxy-2-oxo-2*H*-chromen-4-yl)methyl)-1*H*-1,2,3-triazol-4-yl)methyl)pyrimidine-2,4(1*H*,3*H*)-dione (**9a**)

Pale yellow solid (588 mg, 90%); mp 302 °C with degradation; ^1^H NMR (300 MHz, DMSO-d_6_) *δ* 4.72 (s, 1H), 4.78 (s, 1H), 5.07 (s, 4H), 5.80 (d, *J* 7.9 Hz, 1H), 5.91 (s, 2H), 5.95 (s, 2H), 6.20 (d, *J* 2.2 Hz, 2H), 6.37 (d, *J* 2.2 Hz, 2H), 7.89 (d, *J* 7.9 Hz, 1H), 8.08 (s, 1H), 8.23 (s, 1H); ^13^C NMR (75 MHz, DMSO-d_6_) *δ* 36.0, 43.8, 52.2, 52.3, 62.9, 94.8, 99.6, 100.1, 100.7 (2C), 105.3, 105.5, 125.0 (2C), 125.32 (2C), 142.6, 143.1, 144.6, 151.0, 152.8, 153.0, 156.4 (2C), 158.2, 160.2 (2C), 162.2 (2C), 162.3; HRMS (ESI+) *m*/*z* calcd. for C_30_H_22_N_8_O_10_Na [M+Na]^+^: 677.1351, found: 677.1353.

#### 3.8.2. 1,3-Bis((1-((2-oxo-2*H*-benzo[*h*]chromen-4-yl)methyl)-1*H*-1,2,3-triazol-4-yl)methyl)pyrimidine-2,4(1*H*,3*H*)-dione (**9b**)

Pale yellow solid (586 mg, 85%); mp 193–195 °C; ^1^H NMR (300 MHz, DMSO-d_6_) *δ* 5.06–5.07 (m, 4H), 5.79 (d, *J* 7.9 Hz, 1H), 5.85 (s, 1H), 5.90 (s, 1H), 6.01 (s, 2H), 6.05 (s, 2H), 7.65–7.75 (m, 4H), 7.79–7.91 (m, 5H), 7.99–8.02 (m, 2H), 8.16 (s, 1H), 8.27–8.33 (m, 3H); ^13^C NMR (75 MHz, DMSO-d_6_) *δ* 35.9, 43.8, 49.5, 49.6, 100.6 (2C), 112.7 (2C), 113.0 (2C), 113.1 (2C), 120.2 (2C), 121.6, 122.1, 124.3 (2C), 124.7 (2C), 125.0 (2C), 127.6 (2C), 128.0, 129.0, 134.4, 142.9, 143.3, 144.5 (2C), 150.0, 150.8, 150.9, 151.1, 159.4 (2C), 162.1; HRMS (ESI+) *m*/*z* calcd. for C_38_H_26_N_8_O_6_Na [M+Na]^+^: 713.1868, found: 713.1881.

### 3.9. General Procedure for the Preparation of Inclusion Complex and Dilution

A total of 1 mL of an ethanolic solution containing compounds **7a,b**, **8a,b**, **9a,b**, or **10a,b** (0.004 mol L^−1^) was vigorously stirred with 1 mL of an aqueous solution of HP-β-CD (0.004 mol L^−1^). The resulting mixture was kept under stirring until a clear solution was obtained. The final solution was concentrated under a vacuum to remove the solvent, and the remaining water was removed by lyophilization to give a water-soluble compound of the HP-β-CD complex in a powder form [56]. The stock solutions of the compounds were prepared by dissolving the complexed compounds in 200 µL of DMSO, followed by dissolving in 1800 µL of Dulbecco’s Modified Eagle Medium (DMEM). The stock solution and dilutions were prepared prior to the cytotoxicity assays.

### 3.10. Cells Cultures

The human colon carcinoma (HCT116), human laryngeal tumor (Hep-2), and human lung carcinoma (A549) cell lines, and the non-tumor human keratinocyte cell line (HaCat) were purchased from Rio de Janeiro Cell Bank. The cells were maintained in DMEM supplemented with The, Gibco BRL; Life Technologies, Carlsbad, CA, USA) and 1% penicillin–streptomycin in a humidified 5% CO_2_ atmosphere at 37 °C.

### 3.11. Cell Viability MTT Assay

Cytotoxicity was assayed through the colorimetric microculture MTT assay. Cell suspensions were plated in 96-well plates in triplicate at an initial density of 7 × 10^4^ cells mL^−1^ and incubated at 37 °C for 24 h. After the incubation, the cells were treated with various concentrations of the evaluated compounds (**7a,b**, **8a,b**, **9a,b**, or **10a,b**) and incubated for 24 and 48 h. After the treatment, the medium was removed, and an MTT solution (0.4 mg mL^−1^) was added to each well and further incubated for 2 h at 37 °C. Then, DMSO (100 µL) was added for the solubilization of the formazan crystals, and the absorbance intensities were measured in a microplate reader (SpectraMax M2e, Molecular Devices, Sunnyvale, CA, USA) at 570 nm. The percentage of viable cells was calculated in relation to the control to determine the cytotoxic concentration that reduces 50% of the cell viability (IC_50_).

### 3.12. Biological Target and Toxicity Prediction 

The shared scaffold of uracil, coumarin, and triazole, such as (1-((1-((5,7-dihydroxy-2-oxo-2*H*-chromen-4-yl)methyl)-1*H*-1,2,3-triazol-4-yl)methyl)-3-methylpyrimidine-2,4(1*H*,3*H*)-dione), from the structures of compounds **9a,b** and **10a,b** were submitted for an evaluation of a potential risk to cause mutagenic, tumorigenic, and irritant effects on the reproductive system, with the employment of Osiris Property Explorer^®^ software [43] available free from the web (https://www.organic-chemistry.org/prog/peo/, accessed on 5 August 2020). A Similarity Ensemble Approach (SEA) virtual target screening server [44] was used to predict the potential binding biological targets. Chemical structures of more active compounds and their main fragments were described in smile sequences before performing the software.

### 3.13. Molecular Modeling and Docking Studies

All molecular modeling studies were performed with more active compounds **9a,b** and **10a,b**, aiming to find information that may support the understanding of their biological activity. Geometry optimization and conformational analysis were calculated using Spartan’08^®^ modeling software (V. 1.2.0, Wavefunction Inc., Irvine, CL, USA) based on the DFT-B3LYP/6-311G* method, set in the gas phase. The geometry of compounds was optimized, followed by submitting them to systematic conformational analysis with a torsion angle increment of 30° in the range 0–360°. The lowest energy conformer for the chemical structure was saved in the mol2 file before being used in docking studies. The structure of the human Topo 1-DNA complex encoded by PDB ID: 1K4S [57] was downloaded from the Protein Data Bank (PDB, http://www.rcsb.org/pdb, accessed on 12 August 2020), before performing the docking studies. The cavity of interaction from the Topo 1-DNA model was used in this study. The protein structure was prepared by removing the water molecules and adding polar hydrogens using Autodock Tools (version 1.5.6) [58]. Docking studies were performed using iGemdock software (version 2.1) [49], in which the individual binding poses of compounds were assessed and submitted to dock in the protein. Docking calculations were performed in a drug screening Docking Accuracy Setting with generic evolutionary method (GA) parameters set for population size, generation, and the number of solutions as 200, 70, and 8, respectively, ligand entry energy option active, and Gemdock score function of hydrophobic and electrostatic interactions (1:1 preference). iGemdock software was used to propose the pharmacological pose interactions between the biological receptor and the compound studied.

## 4. Conclusions

In conclusion, we described the synthesis of eight novel compounds that originated from the molecular hybridization of the nucleobases uracil, thymine, or theobromine, with coumarins linked through triazole rings. Among the synthesized compounds, the hybrid compound **9a**, composed of uracil and the coumarin derivative phloroglucinol, showed antitumor activity against colon carcinoma (HCT116) with the lowest value of IC_50_ of 24.19 ± 1.39 µM and a selectivity index of 6.0. The findings of these in vitro results were supported by in silico experiments that, according to the SEA algorithm, indicate that the synthesized compounds show affinity to the Topo 1-DNA complex. Also, molecular docking studies demonstrate that the target compounds **9a,b** and **10a,b** are potential ligands for the Topo 1-DNA complex and possibly act as its inhibitor. Thus, these findings support the potential antitumor activity revealed by these compounds since the Topo 1-DNA complex has been identified as a potential biological target for several anticancer drugs. This protein complex is more expressed in tumor cells than in normal cells. Lastly, in silico toxicity prediction studies have shown that the target compounds possess a low risk of causing theoretical mutagenic and tumorigenic effects compared to H_2_O_2_. Thus, based on the low cytotoxicity shown by compounds **9a,b** and **10a,b**, they can be considered lead scaffolds for further studies.

## Figures and Tables

**Table 1:** Compounds assessed in this study and their cytotoxicity against three different cell lines via the colorimetric MTT assay.

Compound	IC_50_ (µM ± SD) ^a^ (Selectivity Index)							
	HCT116		A549		Hep-2		HaCat	
	24 h	48 h	24 h	48 h	24 h	48 h	24 h	48 h
**7a**	>100	>100	>100	>100	>100	>100	>100	>100
**7b**	>100	>100	>100	>100	>100	>100	>100	>100
**8a**	>100	>100	>100	>100	>100	>100	>100	>100
**8b**	87.58 ± 1.94 (5)	>100	>100	>100	>100	>100	454.1 ± 2.67	>100
**9a**	69.22 ± 1.85 (26)	24.19 ± 1.39 (6)	>100	>100	51.55 ± 1.71 (12)	>100	634.9 ± 2.80	141.6 ± 2.15
**9b**	54.50 ± 1.74 (19)	59.17 ± 1.77 (55)	>100	51.03 ± 1.71 (63)	>100	>100	1049 ± 3.02	3228 ± 3.5
**10a**	78.26 ± 1.89 (4)	>100	>100	>100	56.55 ± 1.75 (5)	>100	307.8 ± 2.49	>100
**10b**	40.98 ± 1.61 (6)	84.74 ± 1.92	>100	>100	>100	>100	263.3 ± 2.42	119.6 ± 2.08
Doxorubicin ^b^	-	1.23 ± 0.05	-^c^	-^c^	-	1.69 ± 0.15	-	39.32 ± 1.59

^a^ SD, standard deviation (*n* = 3). ^b^ Doxorubicin was used as the positive control against HCT116, HEP-2, and HaCat cell lines. HCT116: human colon carcinoma cells; A549: human lung carcinoma cells; Hep-2: human laryngeal tumor cells; HaCat: human keratinocyte. ^c^ IC_50_ for the positive control was not measured for the A459 cell line due to a lack of activity of the target compounds.

**Table 2:** Prediction of theoretical toxicity of **9a,b** and **10a,b** in comparison with H_2_O_2_^a^.

Toxicity Risk	9a	9b	10a	10b	H_2_O_2_
Mutagenic	−	+	−	+	+++
Tumorigenic	−	+	−	+	+++
Irritant	−	−	−	−	+++
Reproductive system	++	−	−	−	+

^a^ The scale of risk of toxicity varies from none (−) to low (+), medium (++), and high (+++) calculated using the Osiris Property Explorer^®^ software [43].

**Table 3:** Main biological target candidates identified by SEA predictions.

Description	*p*-Value	maxTC
Topo 1-DNA complex	3.83 × 10^−31^	0.29
Low molecular weight phosphotyrosine phosphatase	6.667 × 10^−30^	0.30
Testosterone 17-β-dehydrogenase 3	1.506 × 10^−20^	0.37

maxTC: maximum Tanimoto coefficient.

**Table 4:** Calculated total energy (kcal mol^−1^) of compounds **9a,b** and **10a,b** derivatives on the Topo 1-DNA complex ^a^.

Compound	Total Binding Energy ^b^	VDW	H-Bond	Electrostatic Interactions
**9a**	−118.5	−85.6	−32.9	0
**9b**	−113.3	−91.5	−21.8	0
**10a**	−113.7	−68.4	−45.3	0
**10b**	−102.7	−80.9	−21.8	0

^a^ All energy values are given in kcal mol^−1^. ^b^ Total binding energy = VDW + H-Bond + electrostatic interactions. VDW: van der Waal forces; H-bond: hydrogen bond.

**Table 5:** Van der Waals (VDW) and H-bonding (H-bond) pharmacological interactions between ligands **9a,b**, **10a,b**, and the amino acid residues in the binding site of the Topo 1-DNA complex applying the Residues Consensus Analysis ^a^.

Amino Acid Residues	Ligand							
	9a		9b		10a		10b	
	VDW	H-Bond	VDW	H-Bond	VDW	H-Bond	VDW	H-Bond
ARG 364	−5.8	0.0	−4.1	0.0	−1.5	0.0	0.0	0.0
ARG 488	−4.4	−3.4	−3.8	0.0	1.5	−6.3	−4.5	−7.0
LYS 532	−10.2	−4.2	−8.7	0.0	−9.0	−2.2	−1.0	−3.5
ASP 533	1.6	−10.5	−4.7	−8.2	−5.7	−2.5	−0.8	0.0
ILE 535	−16.7	0.0	−15.0	0.0	−4.1	0.0	−1.6	0.0
HIS 632 ^b^	−13.6	−3.5	−21.1	0.0	−4.6	−3.5	−22.5	0.0
THR 718	−3.8	0.0	−5.7	0.0	−6.0	0.0	−5.6	0.0
LEU 721	−9.5	0.0	−2.7	0.0	−13.6	0.0	−19.3	0.0
ASN 722	−1.2	−3.5	−7.3	0.0	−4.4	−3.5	−6.3	−9.9
TYR 723	−0.4	0.0	−2.0	0.0	−4.0	−9.3	−7.3	−4.2
LYS 751	0.0	−7.0	0.0	−3.5	0.0	0.0	0.0	0.0

^a ^All energy values are given in kcal mol^−1^. ^b^ Confirmed by Residues Consensus Analysis. VDW: van der Waal forces; H-bond: hydrogen bond.

## Data Availability

The original contributions presented in the study are included in the article/Supplementary Materials, further inquiries can be directed to the corresponding author.

## Supplementary Materials

The following supporting information can be downloaded at: https://www.mdpi.com/article/10.3390/ph17070956/s1, Copies of ^1^H, ^13^C NMR, and HRMS spectra for all new compounds.
