# Supplementary material for: Novel Coumarin–Nucleobase Hybrids with Potential Anticancer Activity: Synthesis, In Vitro Cell-Based Evaluation, and Molecular Docking
Source: Pharmaceuticals (Basel). 2024 Jul 17;17(7):956. doi: 10.3390/ph17070956 (PMC11279566; doi:10.3390/ph17070956)
Supplement: Supplementary file 1 [file pharmaceuticals-17-00956-s001.zip › Supplementary Material_Revised Version.pdf]

Supplementary material

Novel coumarin-nucleobase hybrids with potential anticancer activity: Synthesis, *in-vitro* cell-based evaluation, and molecular docking

Maiara C. de Moraes [1,2], Rafaele Frassini [1], Mariana Roesch-Ely [1], Favero R. Paula [3] and Thiago Barcellos [1,*]

[1] Universidade de Caxias do Sul, Francisco Getúlio Vargas St., 1130, 95070-560, Caxias do Sul, RS, Brazil.
[2] Instituto Federal de Educação, Ciência e Tecnologia do Rio Grande do Sul – Campus Caxias do Sul, Avelino Antônio de Souza, 1730, 95043-700, Caxias do Sul, RS, Brazil.
[3] Laboratório de Desenvolvimento e Controle de Qualidade em Medicamentos, Universidade Federal do Pampa, Campus Uruguaiana, BR 472, Km 592, 97508-000, Uruguaiana, RS, Brazil.
* Correspondence: thiago.barcellos@ucs.br; Tel.: +55 (54) 32182668

Contents:

1. Copies of ^{1}H and ^{13}C NMR spectra for all new compounds……………………………….……...S2
2. ^{1}H NMR spectra of (a) (2-Hydroxypropyl)-β-cyclodextrin (HP-β-CD), (b) compound **9a**, and, (c) the inclusion complex resulting from the mixture of the HP-β-CD and compound **9a**…………………………………………………………………………………………….S15
3. Copied of HRMS analysis for the compounds **7a**, **7b**, **8a**, **8b**, **9a**, **9b**, **10a**, and **10b**………..……S16

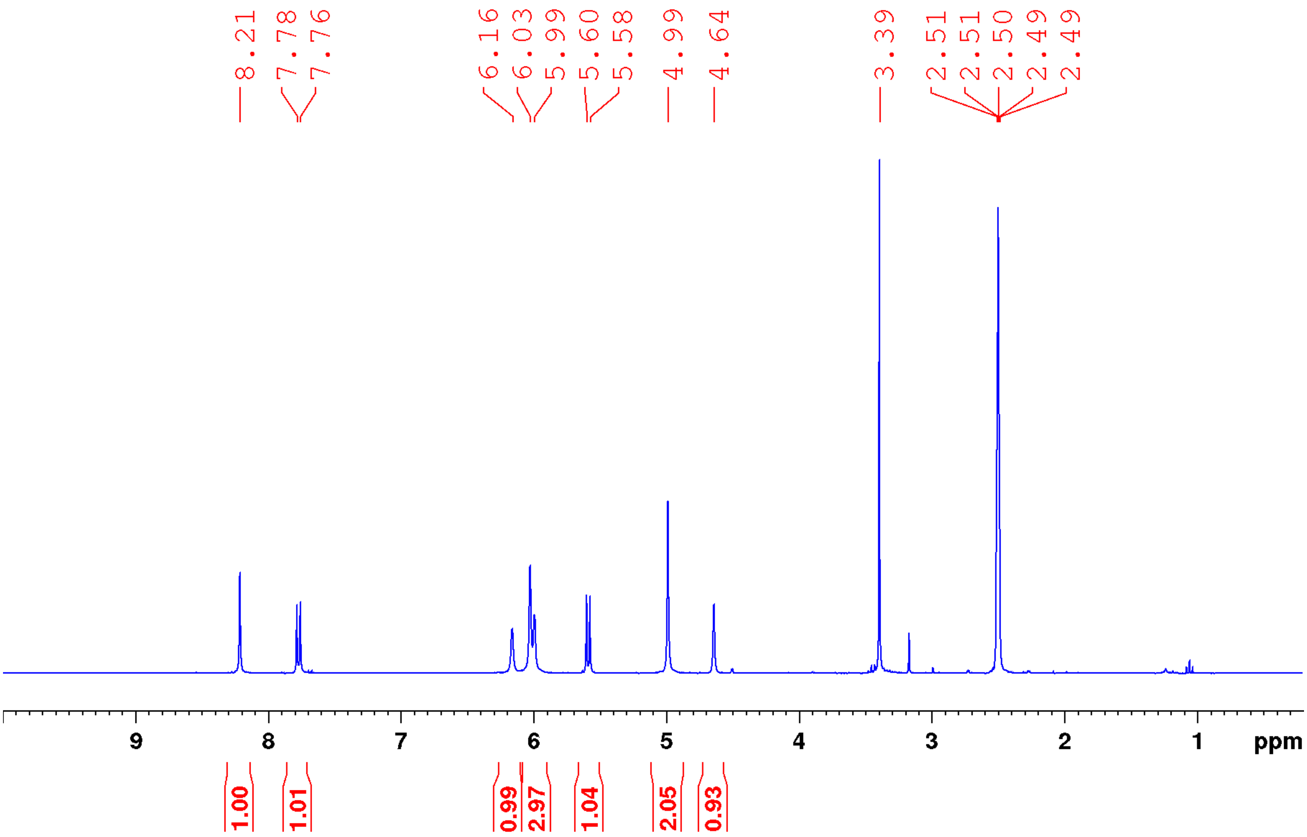

Figure S1. ^{1}H NMR spectrum (DMSO-d_6, 300 MHz) of 1-((1-((5,7-dihydroxy-2-oxo-2*H*-chromen-4-yl)methyl)-1*H*-1,2,3-triazol-4-yl)methyl)pyrimidine-2,4(1*H*,3*H*)-dione (**7a**).

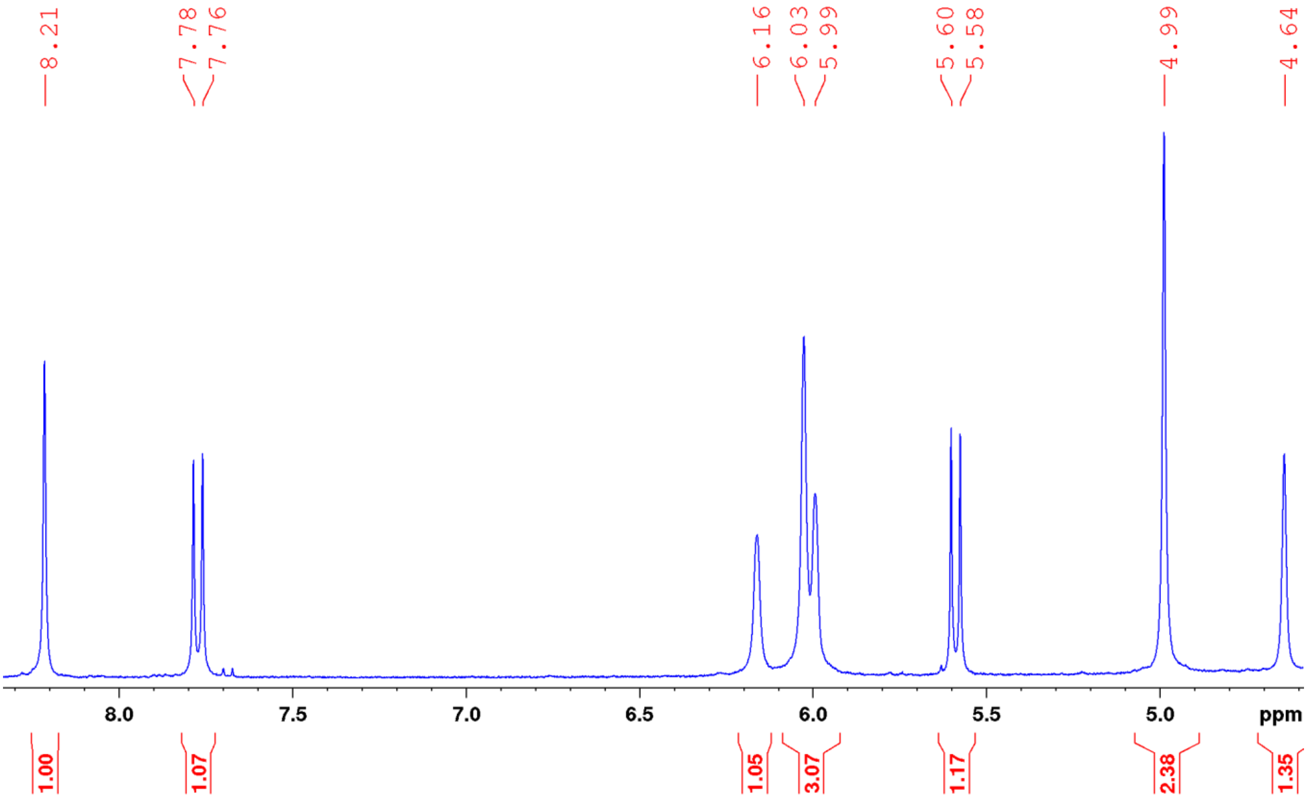

Figure S2. Expansion of the ^{1}H NMR spectrum (DMSO-d_6, 300 MHz) of compound **7a**.

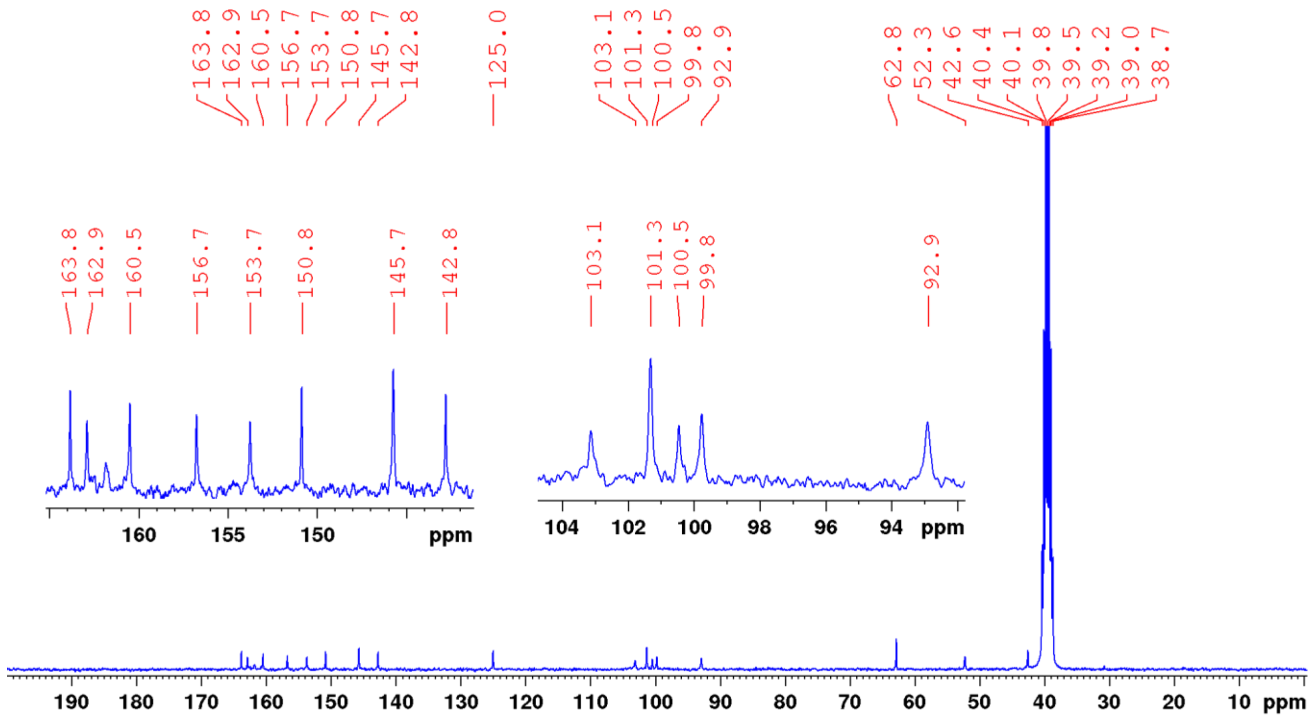

Figure S3. ^{13}C NMR spectrum (DMSO-d6, 75 MHz) of 1-((1-((5,7-dihydroxy-2-oxo-2*H*-chromen-4-yl)methyl)-1*H*-1,2,3-triazol-4-yl)methyl)pyrimidine-2,4(1*H*,3*H*)-dione (**7a**).

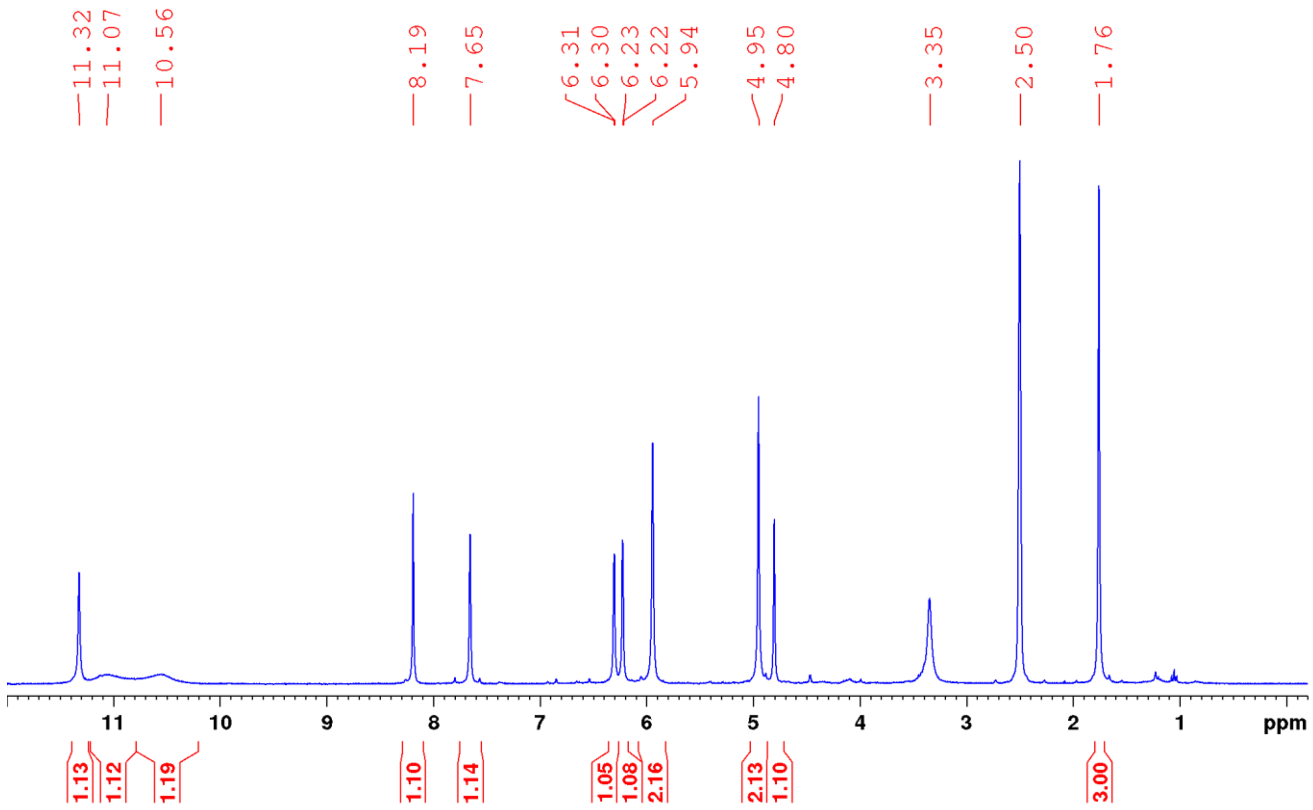

Figure S4. ^{1}H NMR spectrum (DMSO-d6, 300 MHz) of 1-((1-((5,7-dihydroxy-2-oxo-2*H*-chromen-4-yl)methyl)-1*H*-1,2,3-triazol-4-yl)methyl)-5-methylpyrimidine-2,4(1*H*,3*H*)-dione (**7b**).

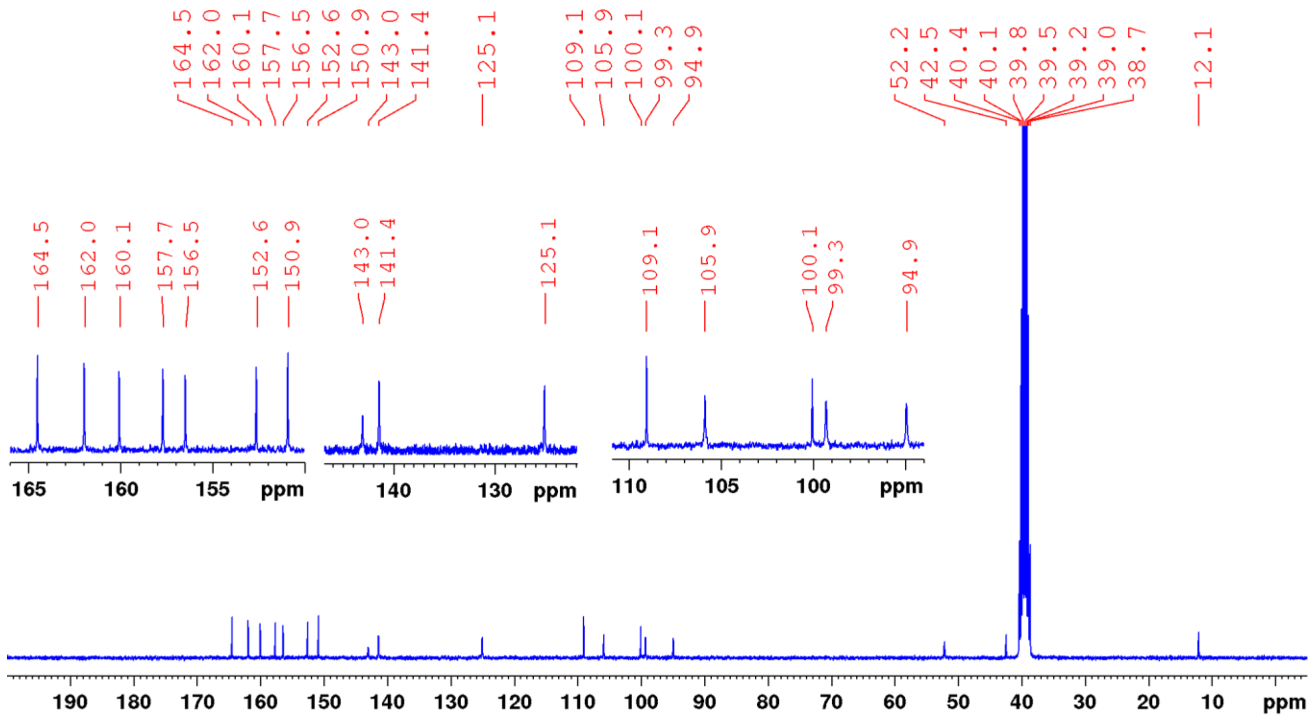

Figure S5. ^{13}C NMR spectrum (DMSO-d_6, 75 MHz) of 1-((1-((5,7-dihydroxy-2-oxo-2*H*-chromen-4-yl)methyl)-1*H*-1,2,3-triazol-4-yl)methyl)-5-methylpyrimidine-2,4(1*H*,3*H*)-dione (**7b**).

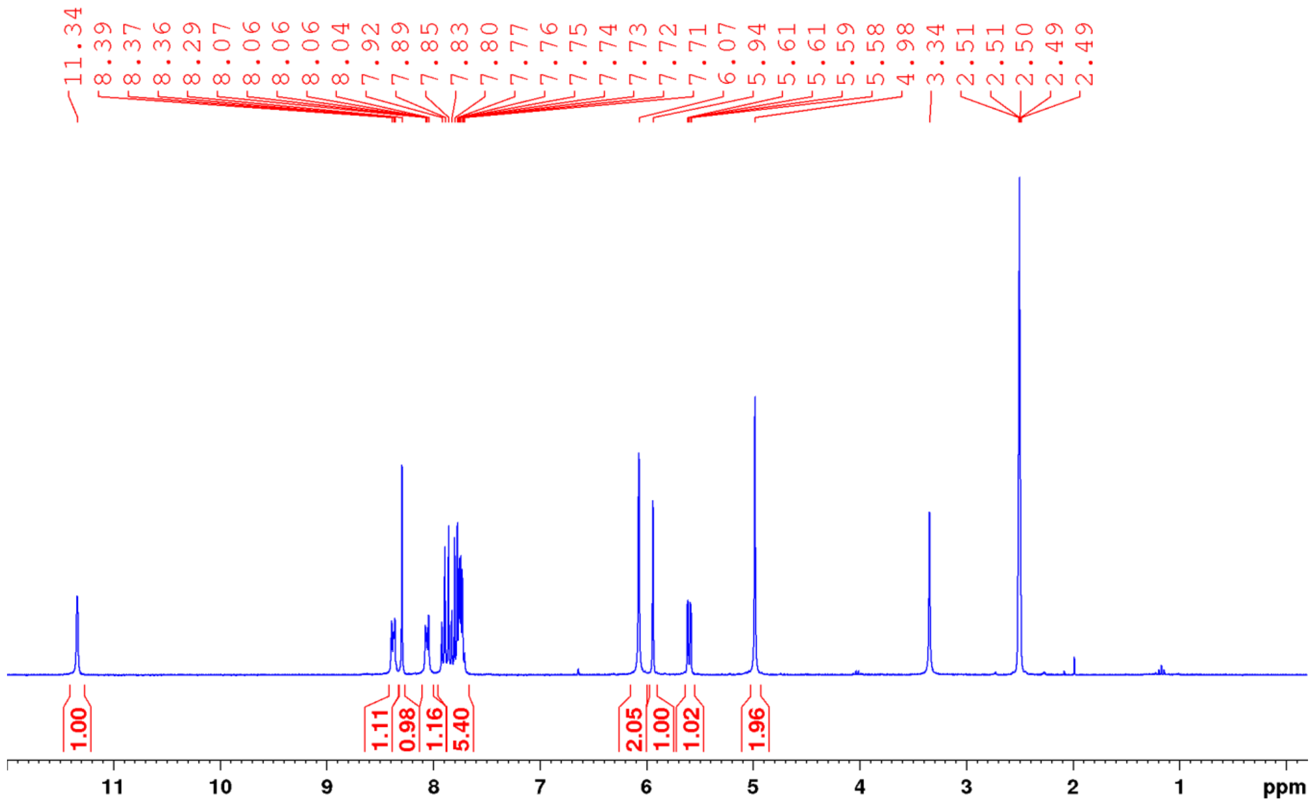

Figure S6. ^1H NMR spectrum (DMSO-d_6, 300 MHz,) of 1-((1-((2-oxo-2H-benzo[*h*]chromen-4-yl)methyl)-1*H*-1,2,3-triazol-4-yl)methyl)pyrimidine-2,4(1*H*,3*H*)-dione (**8a**).

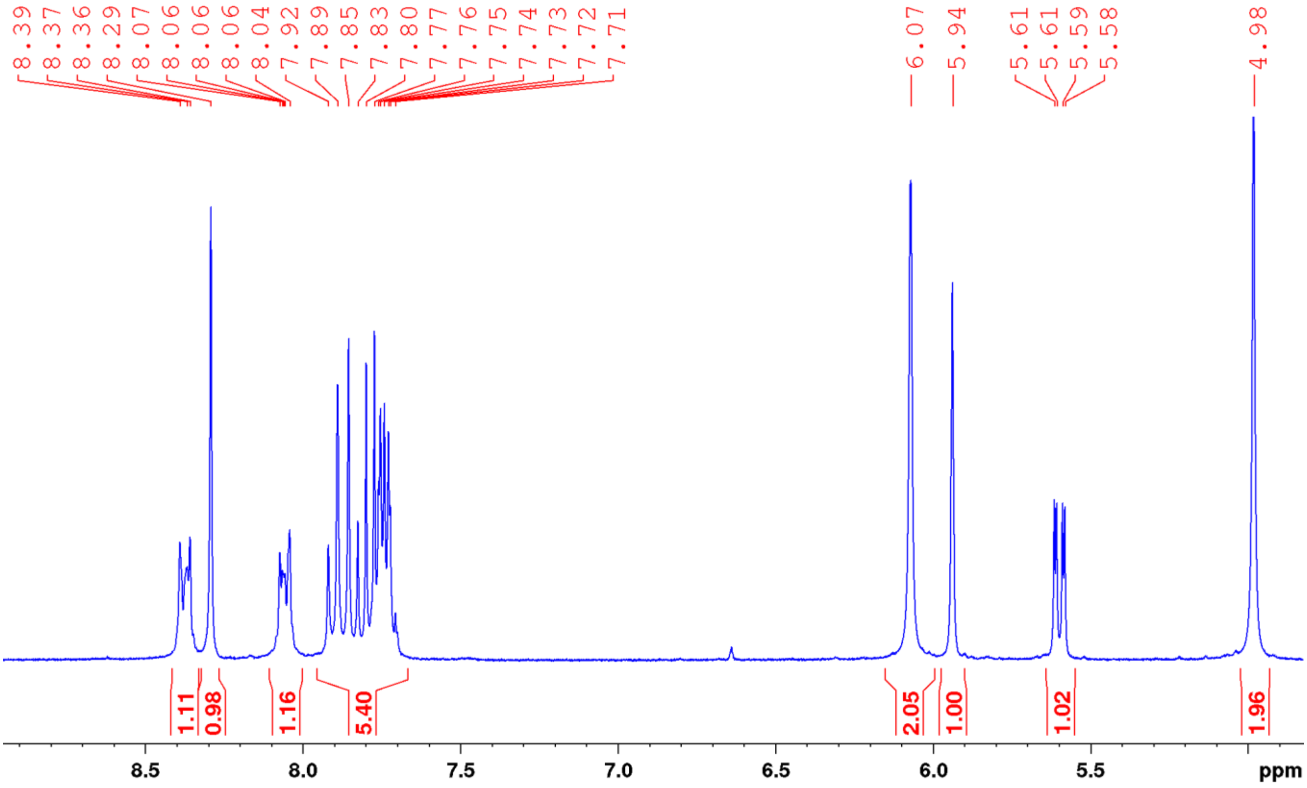

Figure S7. Expansion of the ^{1}H NMR spectrum (300 MHz, DMSO-d_6) of compound **8a**.

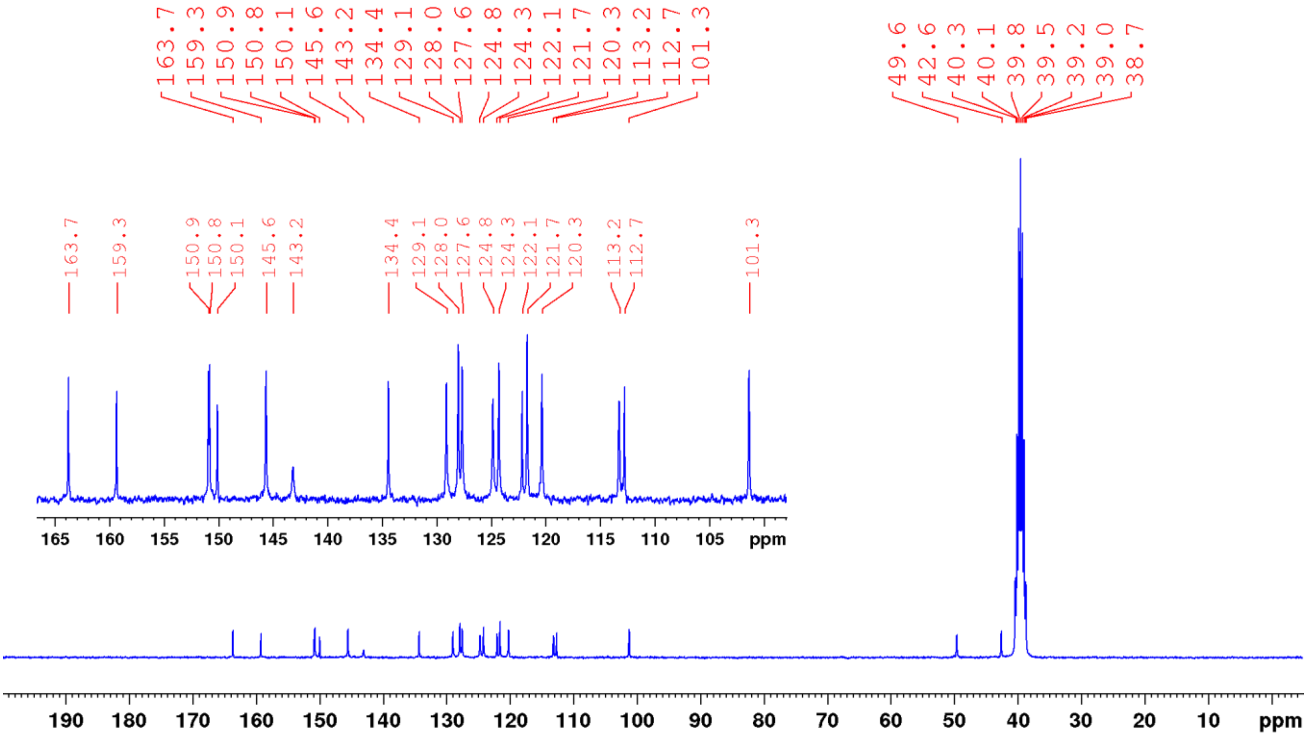

Figure S8. ^{13}C NMR spectrum (DMSO-d_6, 75 MHz) of 1-((1-((2-oxo-2H-benzo[*h*]chromen-4-yl)methyl)-1*H*-1,2,3-triazol-4-yl)methyl)pyrimidine-2,4(1*H*,3*H*)-dione (**8a**).

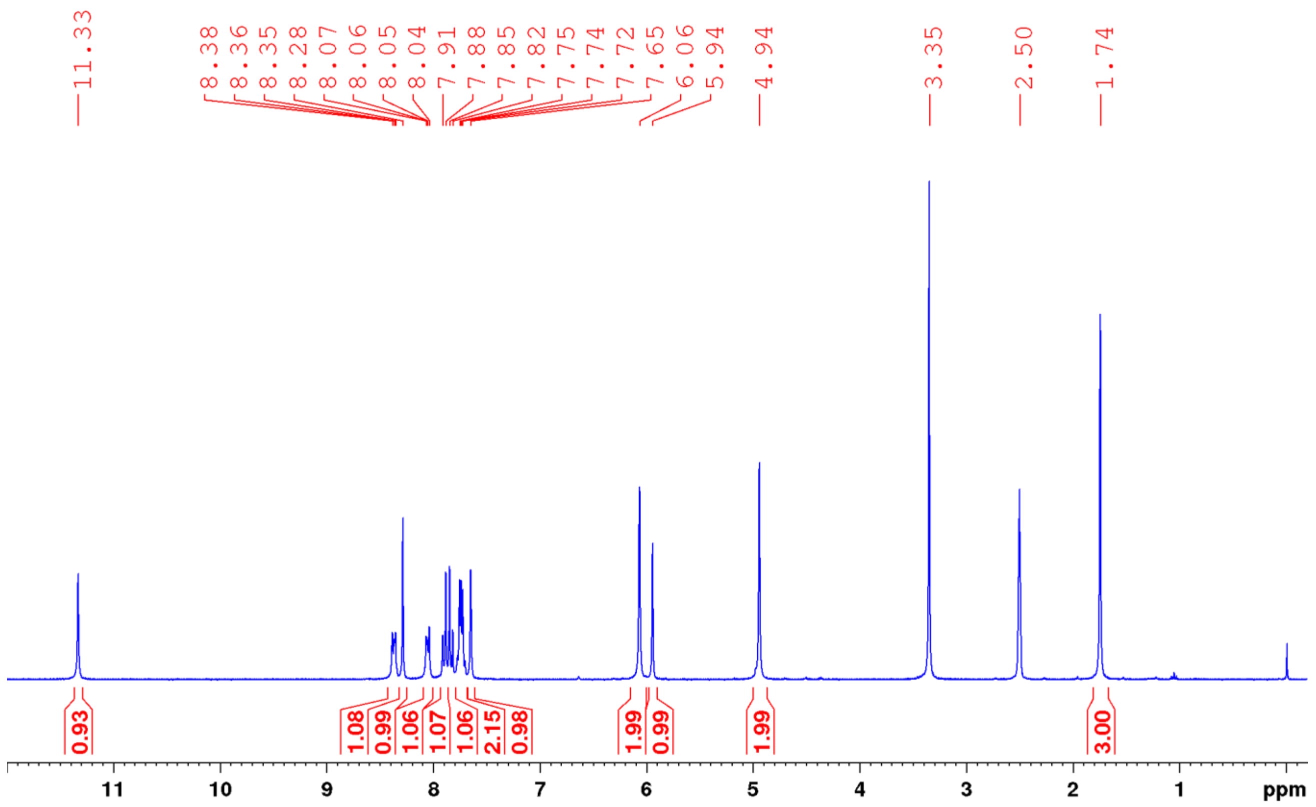

Figure S9. ¹H NMR spectrum (DMSO-d₆, 300 MHz) of 1-((1-((2-oxo-2H-benzo[*h*]chromen-4-yl)methyl)-1*H*-1,2,3-triazol-4-yl)methyl)-5-methylpyrimidine-2,4(1*H*,3*H*)-dione (**8b**).

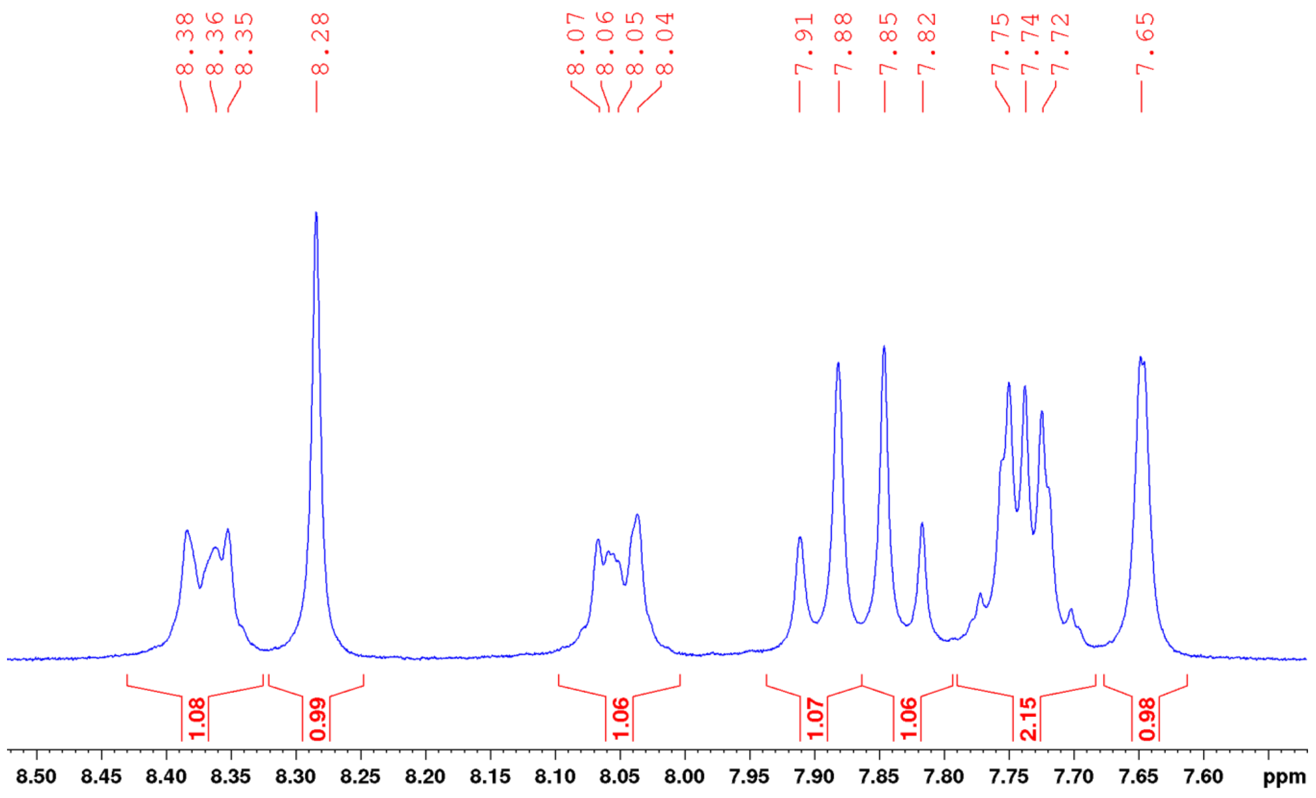

Figure S10. Expansion of the ¹H NMR spectrum (DMSO-d₆, 300 MHz) of compound (**8b**).

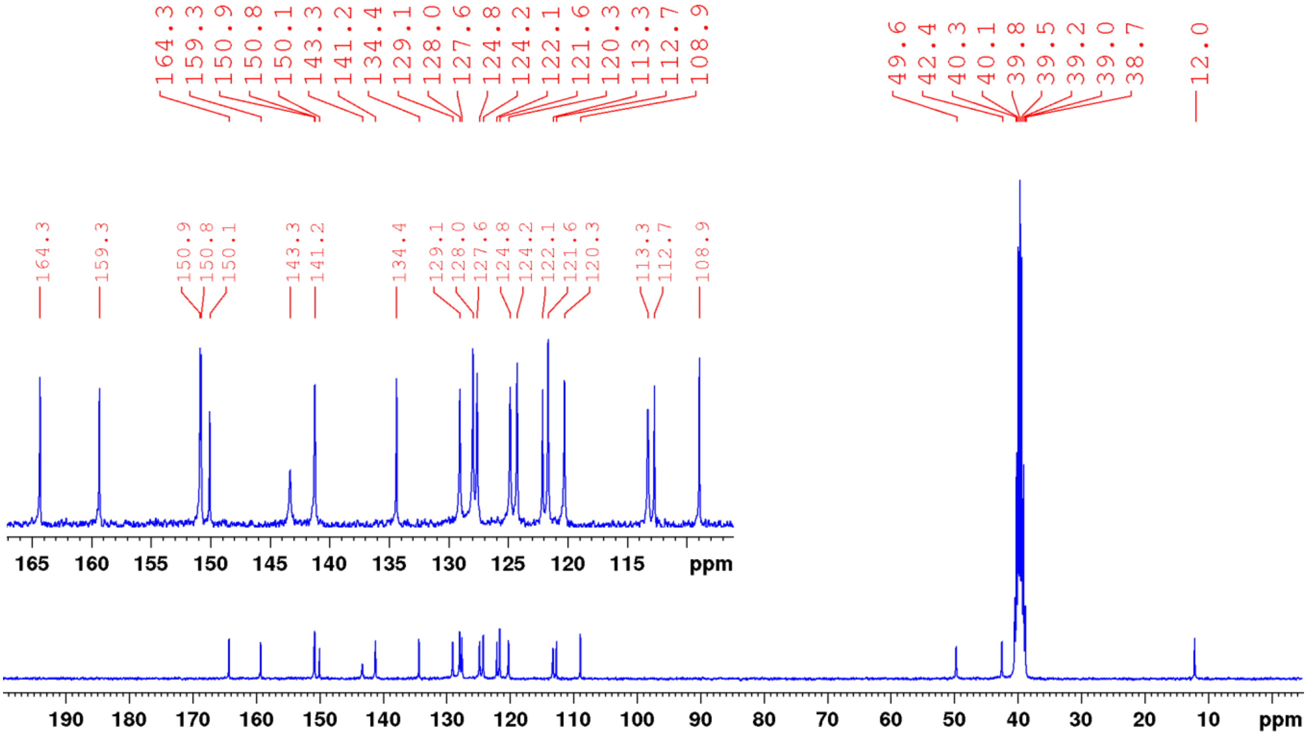

Figure S11. ^{13}C NMR spectrum (DMSO-d_6, 75 MHz) of 1-((1-((2-oxo-2H-benzo[*h*]chromen-4-yl)methyl)-1*H*-1,2,3-triazol-4-yl)methyl)-5-methylpyrimidine-2,4(1*H*,3*H*)-dione (**8b**).

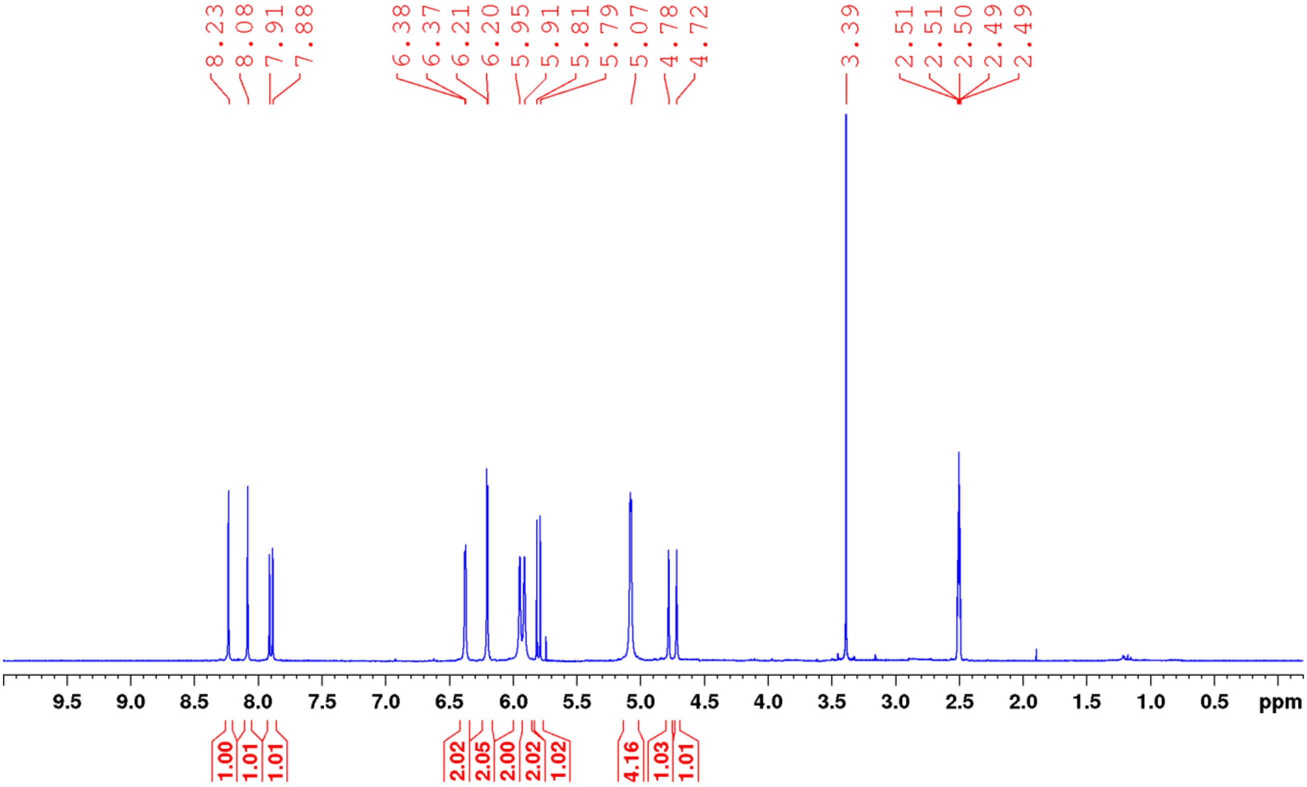

Figure S12. ^{1}H NMR spectrum (DMSO-d_6, 300 MHz) of 1,3-bis((1-((5,7-dihydroxy-2-oxo-2*H*-chromen-4-yl)methyl)-1*H*-1,2,3-triazol-4-yl)methyl)pyrimidine-2,4(1*H*,3*H*)-dione (**9a**).

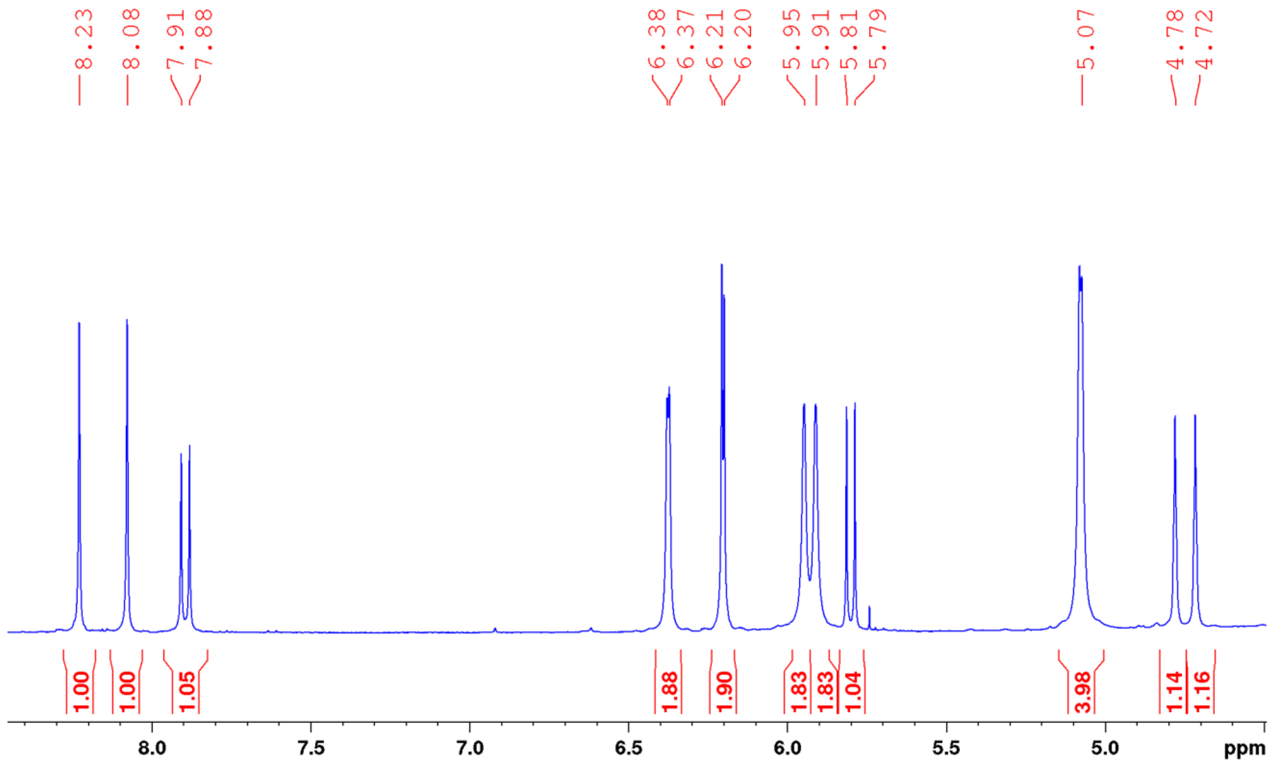

Figure S13. Expansion of the ^{1}H NMR spectrum (DMSO-d6, 300 MHz) of compound **9a**.

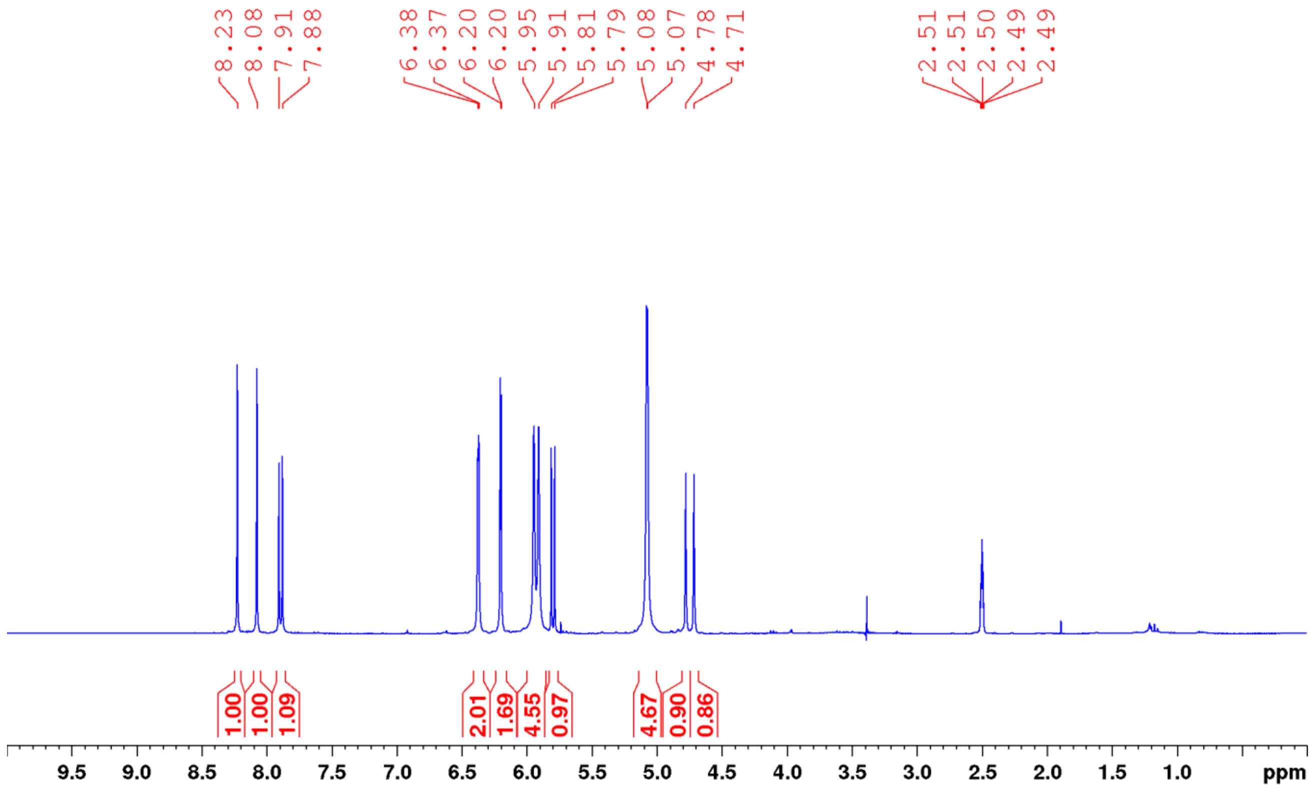

Figure S14. ^{1}H NMR spectrum (DMSO-d6, 300 MHz) of 1,3-bis((1-((5,7-dihydroxy-2-oxo-2*H*-chromen-4-yl)methyl)-1*H*-1,2,3-triazol-4-yl)methyl)pyrimidine-2,4(1*H*,3*H*)-dione (**9a**) with suppression of the water signal.

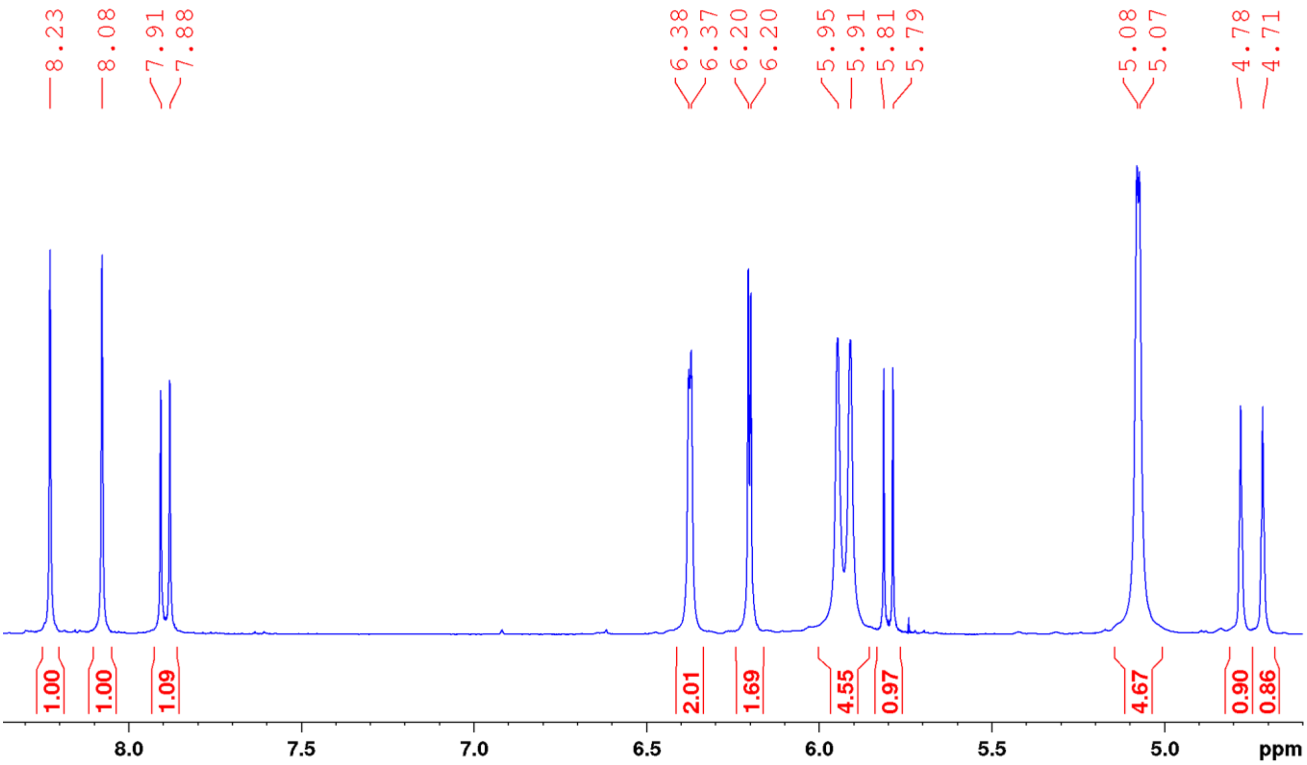

Figure S15. Expansion of the ^{1}H NMR spectrum (DMSO-d6, 300 MHz) of compound **9a** with suppression of the water signal.

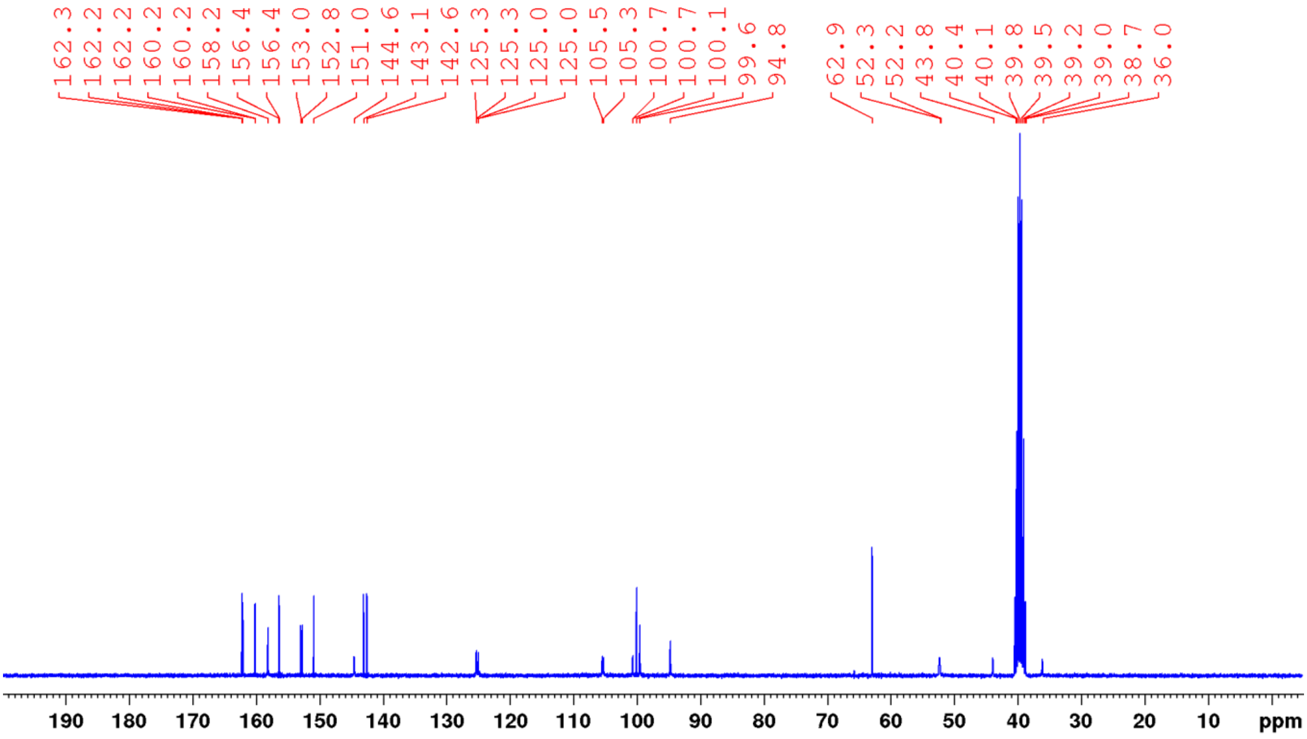

Figure S16. ^{13}C NMR spectrum (DMSO-d6, 75 MHz) of 1,3-bis((1-((5,7-dihydroxy-2-oxo-2*H*-chromen-4-yl)methyl)-1*H*-1,2,3-triazol-4-yl)methyl)pyrimidine-2,4(1*H*,3*H*)-dione (**9a**).

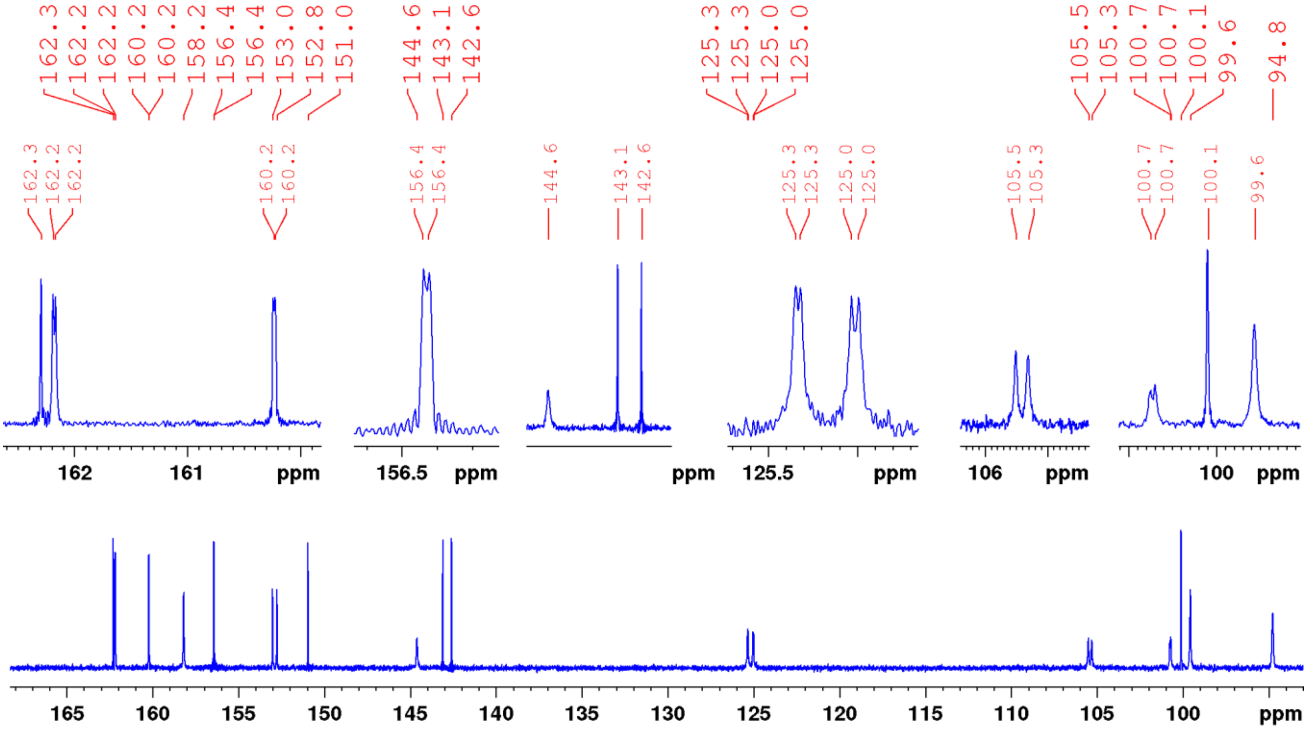

Figure S17. Expansion of the 13C NMR spectrum (DMSO-d6, 75 MHz) of compound **9a**.

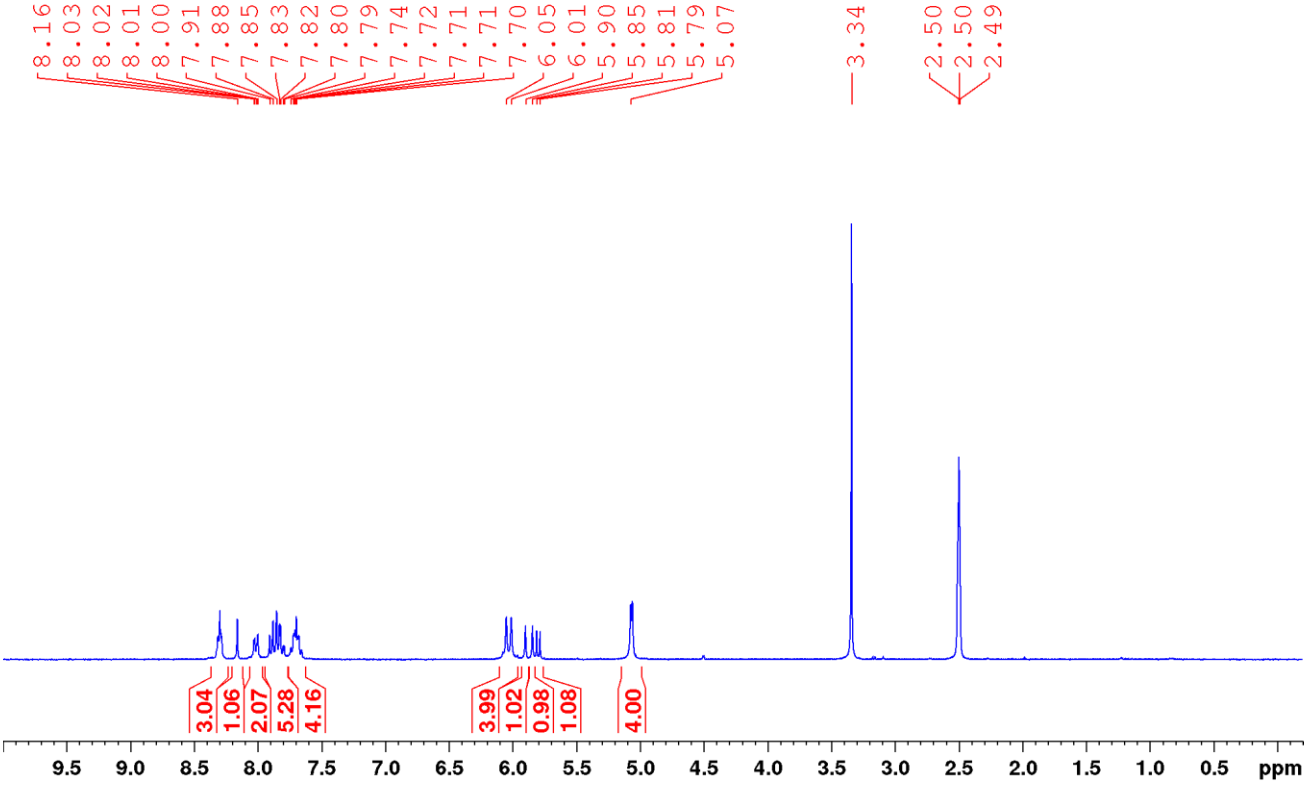

Figure S18. ^{1}H NMR spectrum (DMSO-d$_6$, 300 MHz) of 1,3-bis((1-((2-oxo-2H-benzo[*h*]chromen-4 yl)methyl)-1*H*-1,2,3-triazol-4-yl)methyl)pyrimidine-2,4(1*H*,3*H*)-dione (**9b**).

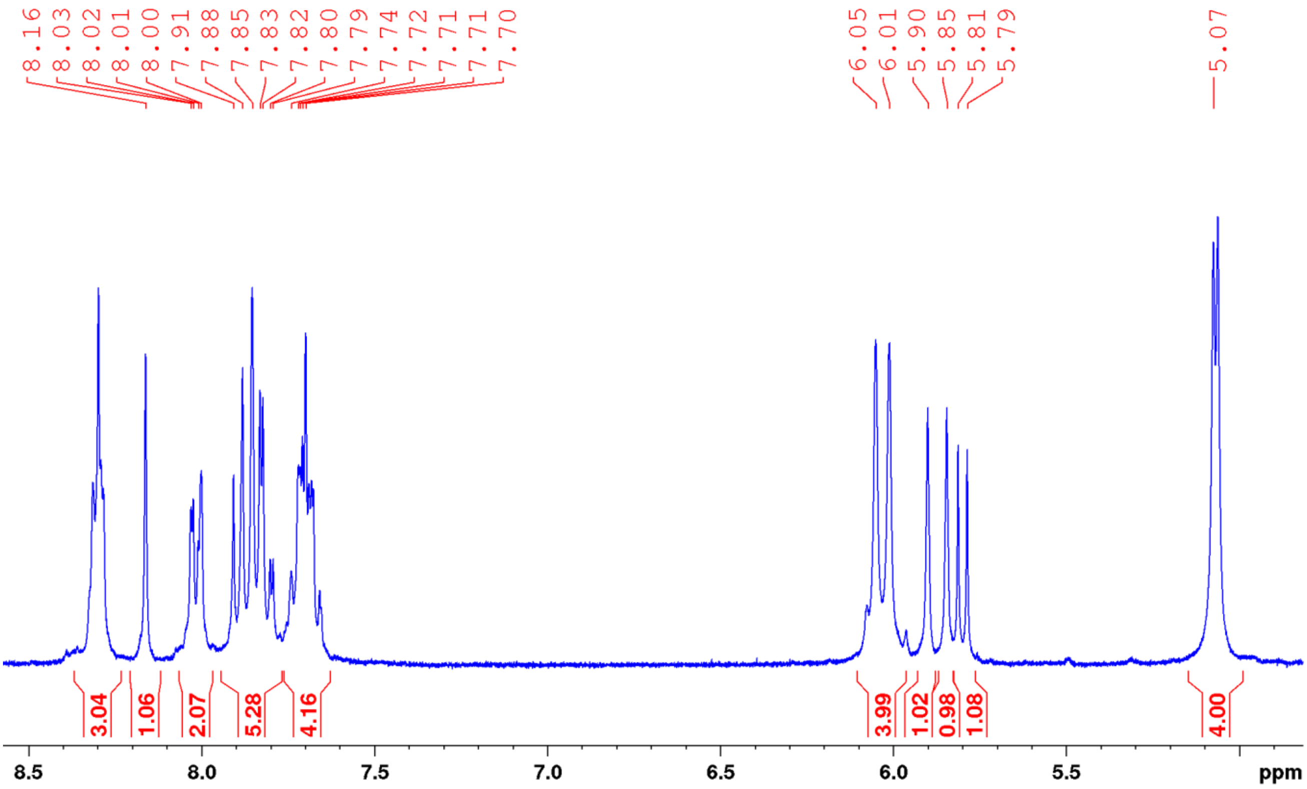

Figure S19. Expansion of the ^{1}H NMR spectrum (DMSO-d$_6$, 300 MHz) of compound **9b**.

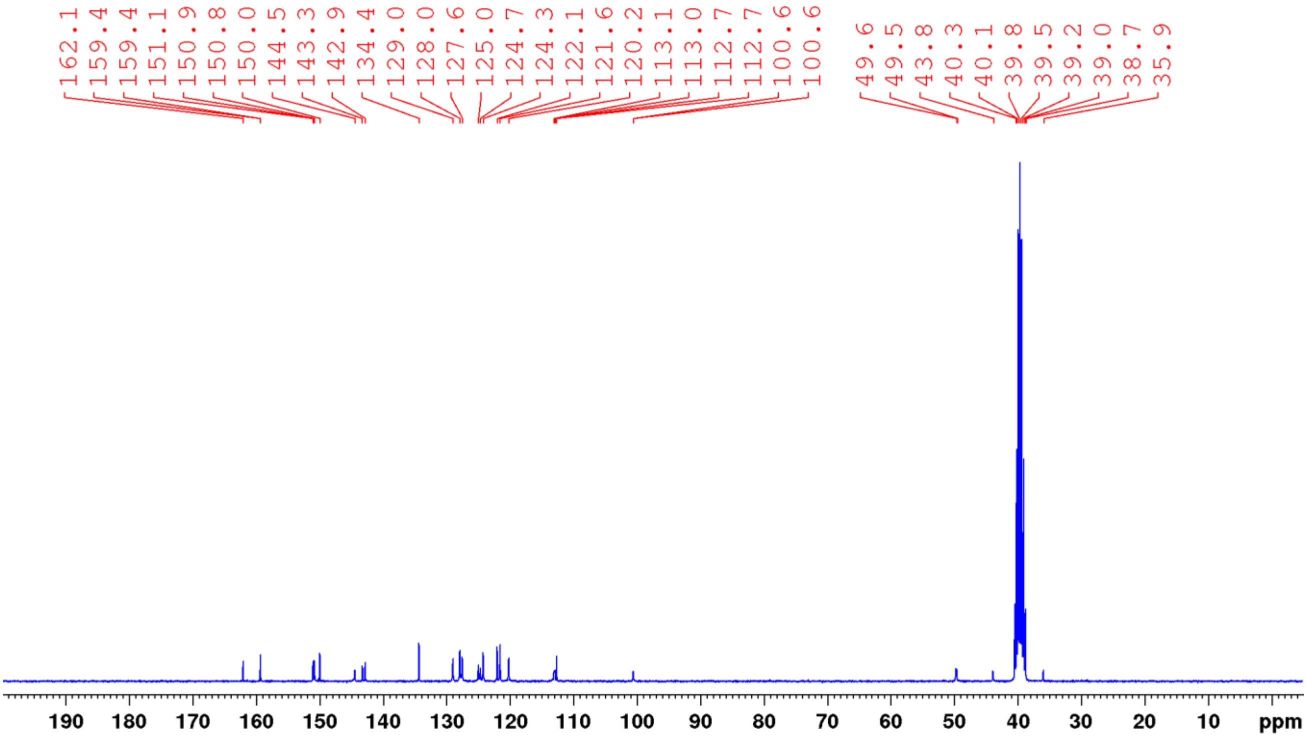

Figure S20. ^{13}C NMR spectrum (DMSO-d$_6$, 75 MHz) of 1,3-bis((1-((2-oxo-2H-benzo[*h*]chromen-4-yl)methyl)-1*H*-1,2,3-triazol-4-yl)methyl)pyrimidine-2,4(1*H*,3*H*)-dione (**9b**).

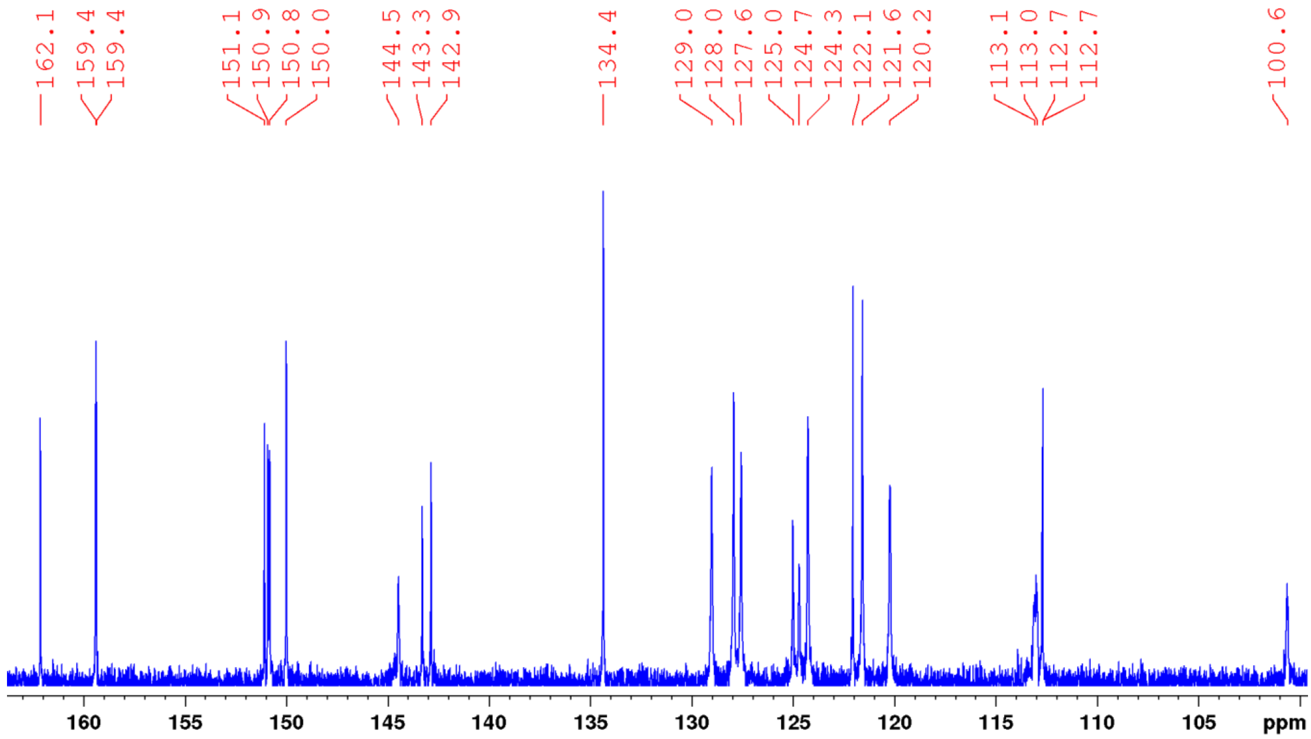

Figure S21. Expansion of the ^{13}C NMR spectrum (DMSO-d$_6$, 75 MHz) of compound **9b**.

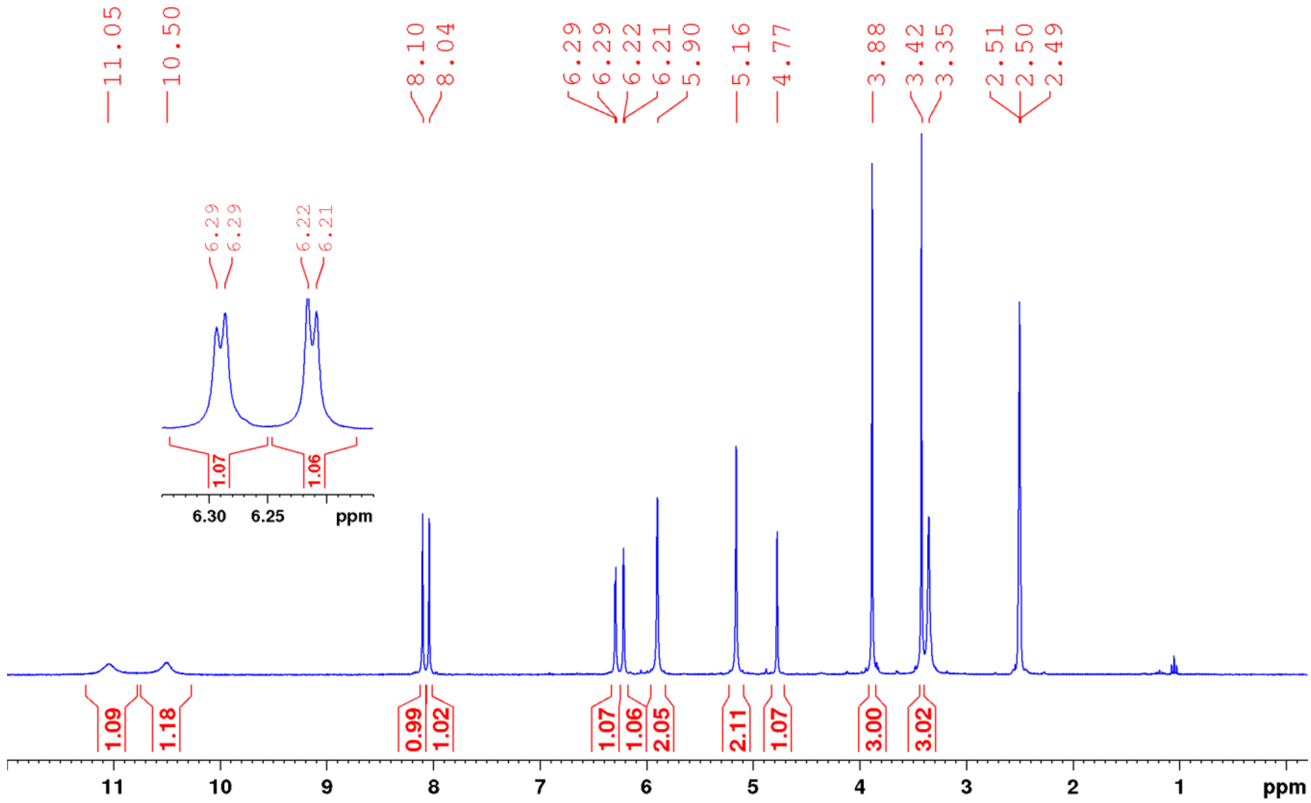

Figure S22. ^1H NMR spectrum (DMSO-d$_6$, 300 MHz) of 1-((1-((5,7-dihydroxy-2-oxo-2*H*-chromen-4-yl)methyl)-1*H*-1,2,3-triazol-4-yl)methyl)-3,7-dimethyl-3,7-dihydro-1*H*-purine-2,6-dione (**10a**).

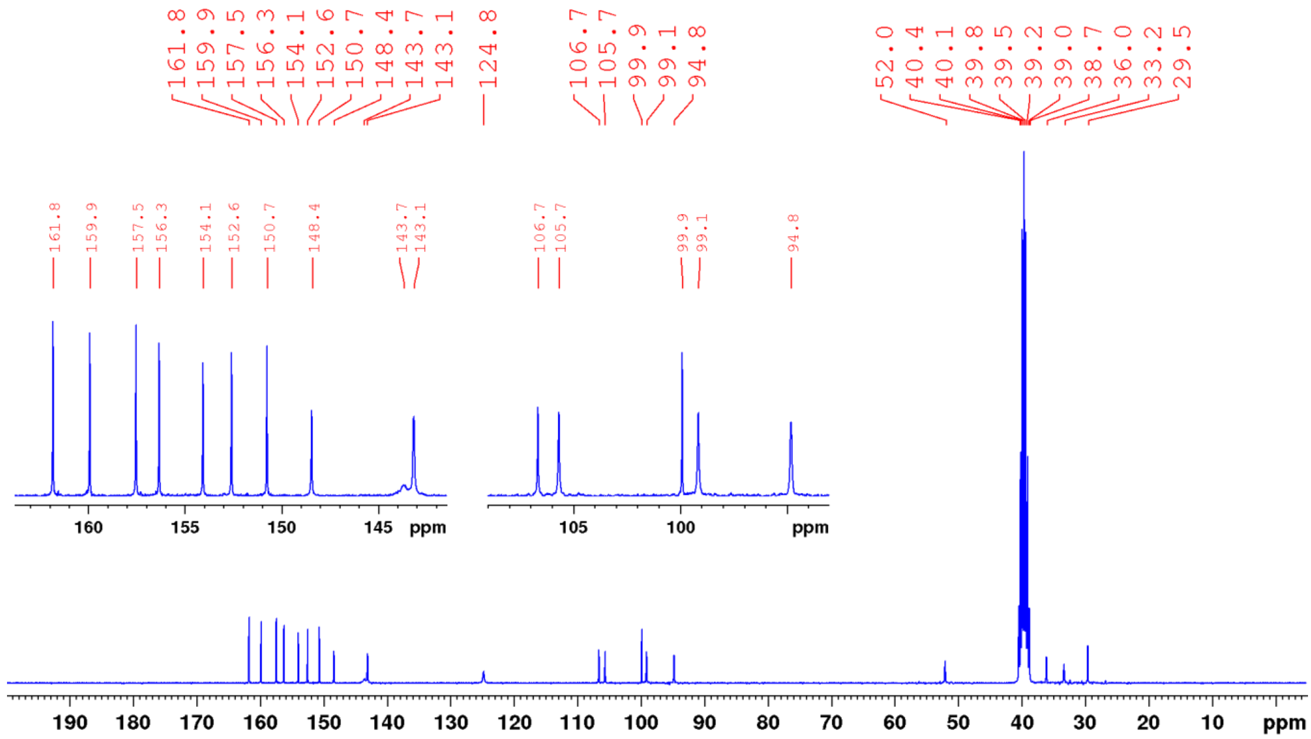

Figure S23. ^{13}C NMR spectrum (DMSO-d6, 75 MHz) of 1-((1-((5,7-dihydroxy-2-oxo-2*H*-chromen-4-yl)methyl)-1*H*-1,2,3-triazol-4-yl)methyl)-3,7-dimethyl-3,7-dihydro-1*H*-purine-2,6-dione (**10a**).

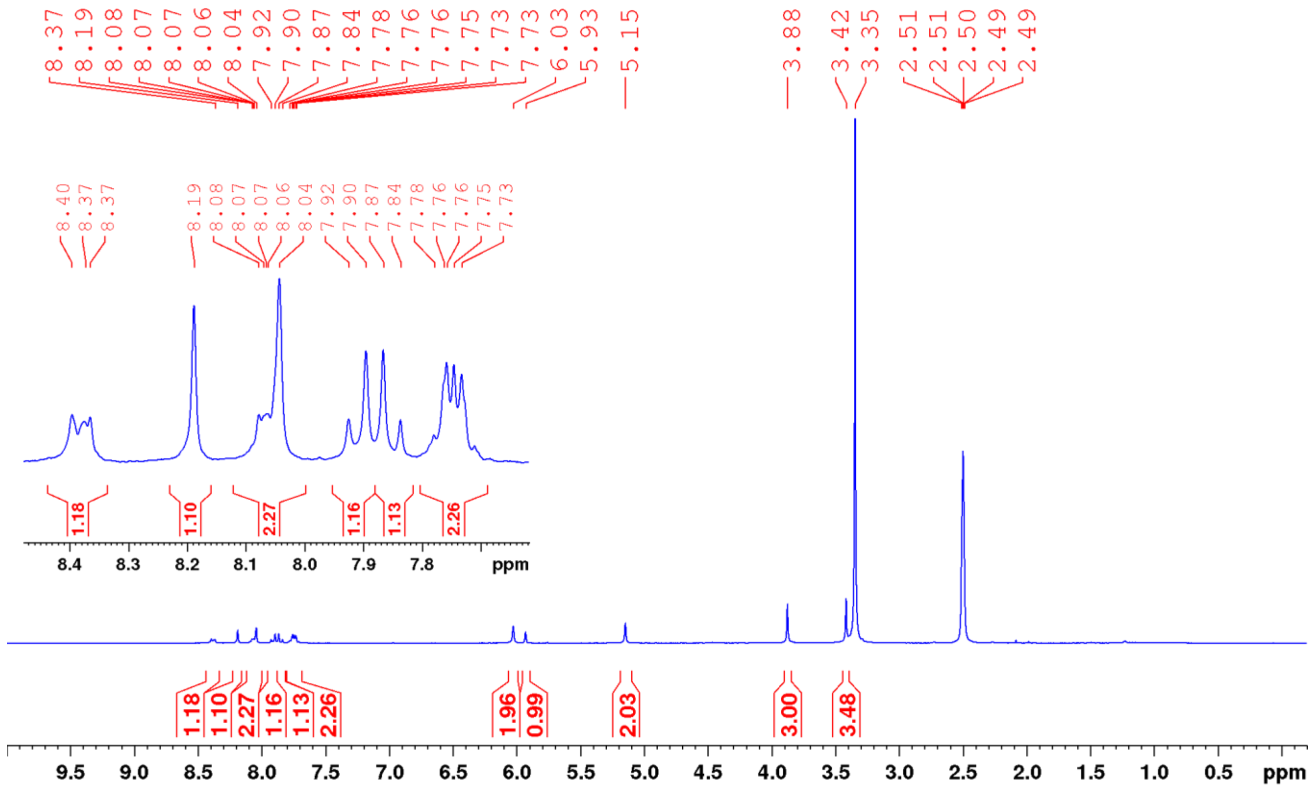

Figure S24. ^{1}H NMR spectrum (DMSO-d6, 300 MHz) of 3,7-dimethyl-1-((1-((2-oxo-2*H*-benzo[*h*]chromen-4-yl)methyl)-1*H*-1,2,3-triazol-4-yl)methyl)-3,7-dihydro-1*H*-purine-2,6-dione (**10b**).

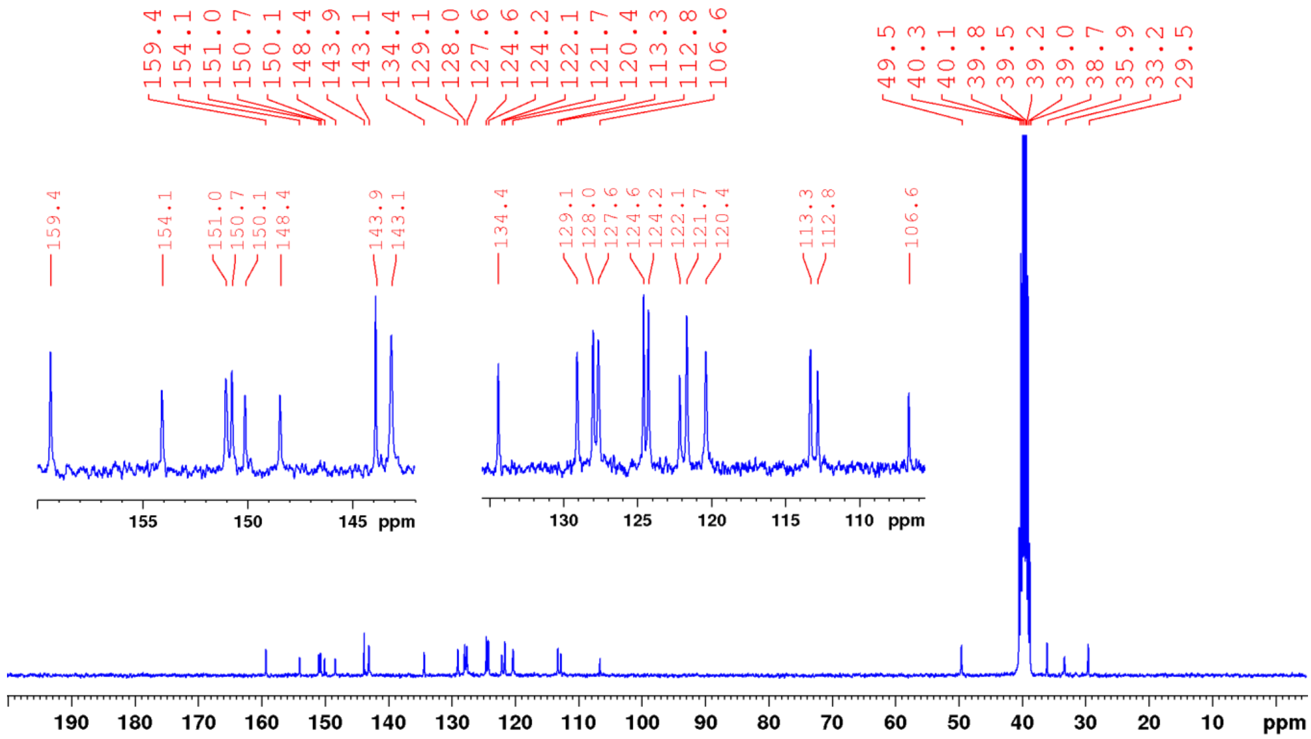

Figure S25. ^{13}C NMR spectrum (DMSO-d_6, 75 MHz) of 3,7-dimethyl-1-((1-((2-oxo-2H-benzo[*h*]chromen-4-yl)methyl)-1*H*-1,2,3-triazol-4-yl)methyl)-3,7-dihydro-1*H*-purine-2,6-dione (**10b**).

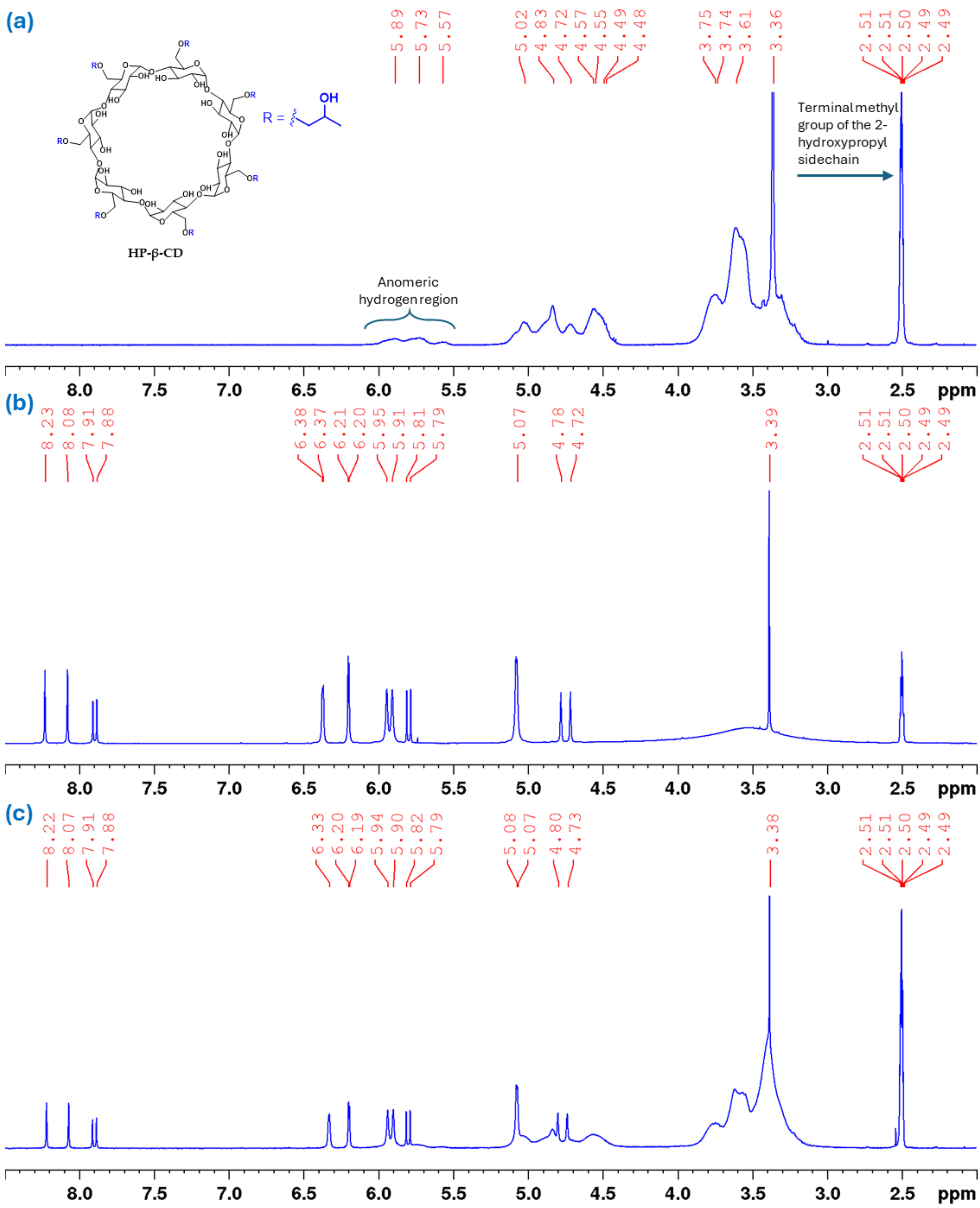

Figure S26. ^{1}H NMR spectra of (a) (2-Hydroxypropyl)-β-cyclodextrin (HP-β-CD), (b) compound **9a**, and, (c) the inclusion complex resulting from the mixture of the HP-β-CD and compound **9a**.

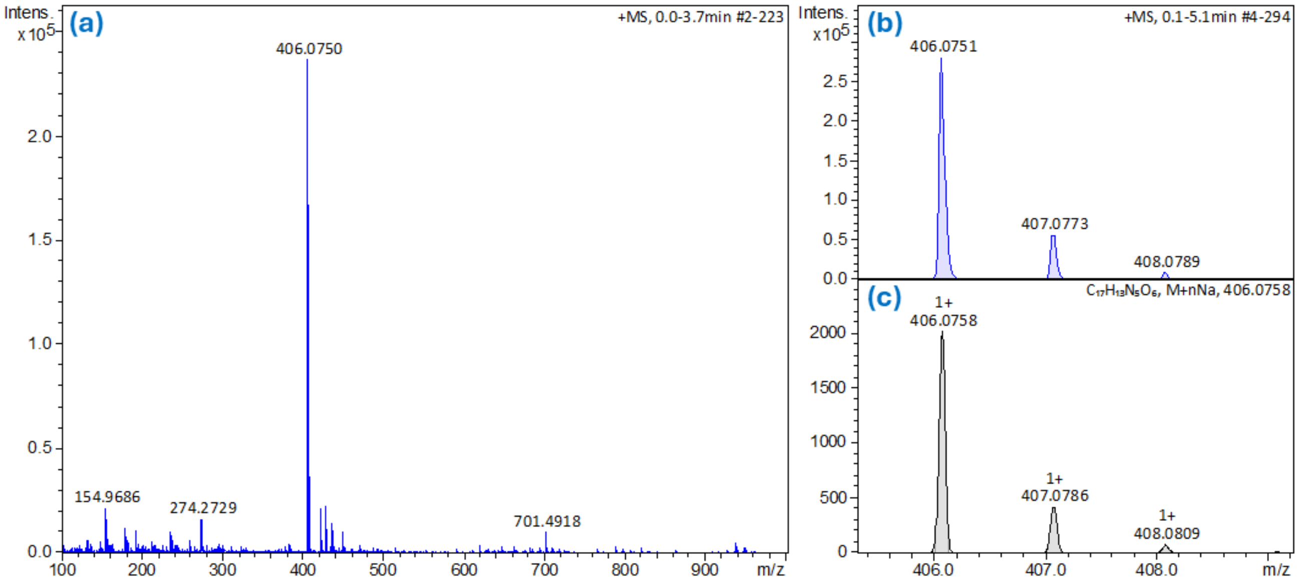

Figure S27. (a) HRMS spectrum (ESI-QToF) of compound **7a** (a), the expanded regium of the spectrum detailing the sodium-cationized molecule $[M+Na]^+$ and, (c) the simulated spectrum for $C_{17}H_{13}N_5O_6Na$ $[M+Na]^+$.

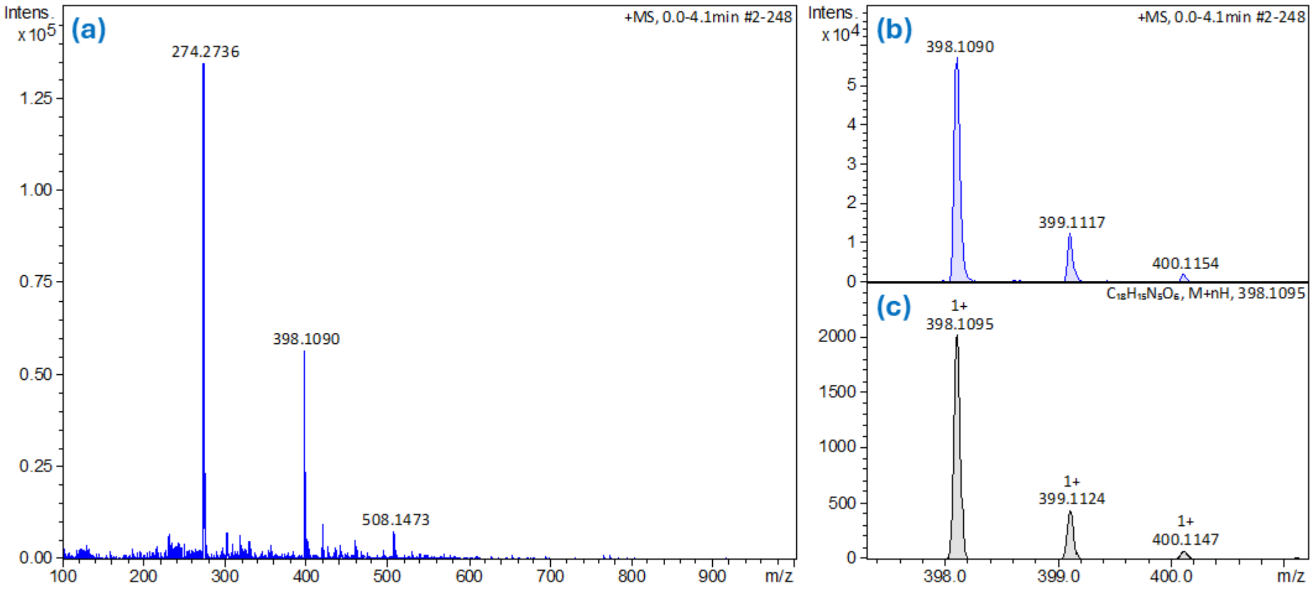

Figure S28. (a) HRMS spectrum (ESI-QToF) of compound **7b** (a), the expanded regium of the spectrum detailing the protonated molecule $[M+H]^+$ and, (c) the simulated spectrum for $C_{18}H_{16}N_5O_6$ $[M+H]^+$.

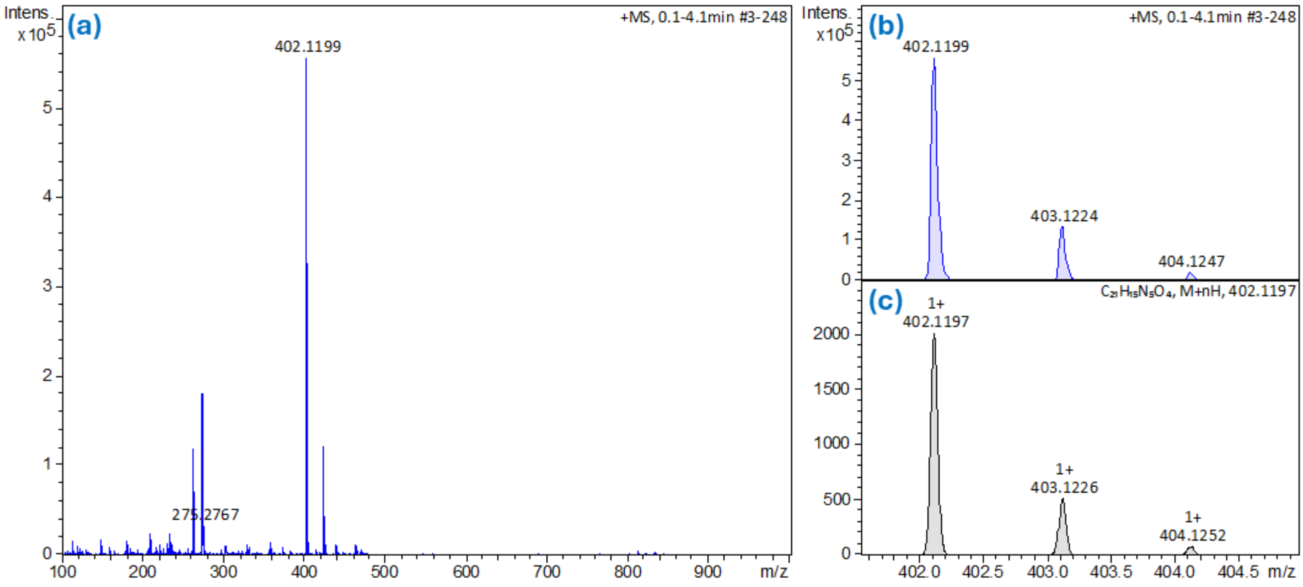

Figure S29. (a) HRMS spectrum (ESI-QToF) of compound **8a** (a), the expanded regium of the spectrum detailing the protonated molecule $[M+H]^+$ and, (c) the simulated spectrum for $C_{21}H_{16}N_5O_4$ $[M+H]^+$.

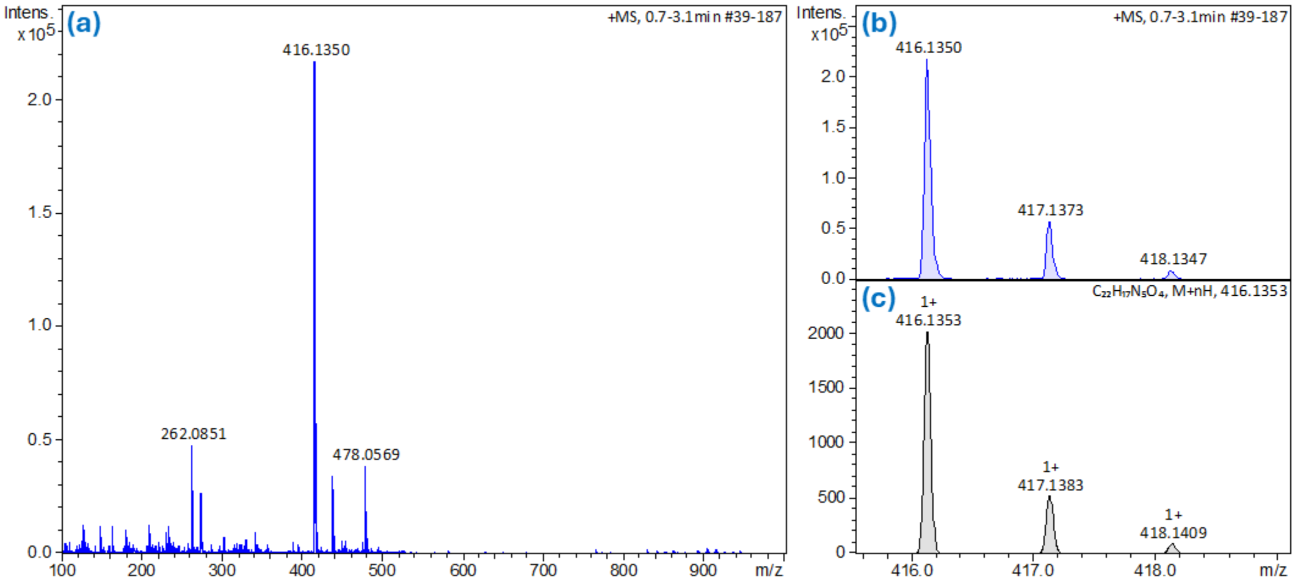

Figure S30. (a) HRMS spectrum (ESI-QToF) of compound **8b** (a), the expanded regium of the spectrum detailing the protonated molecule $[M+H]^+$ and, (c) the simulated spectrum for $C_{22}H_{18}N_5O_4$ $[M+H]^+$.

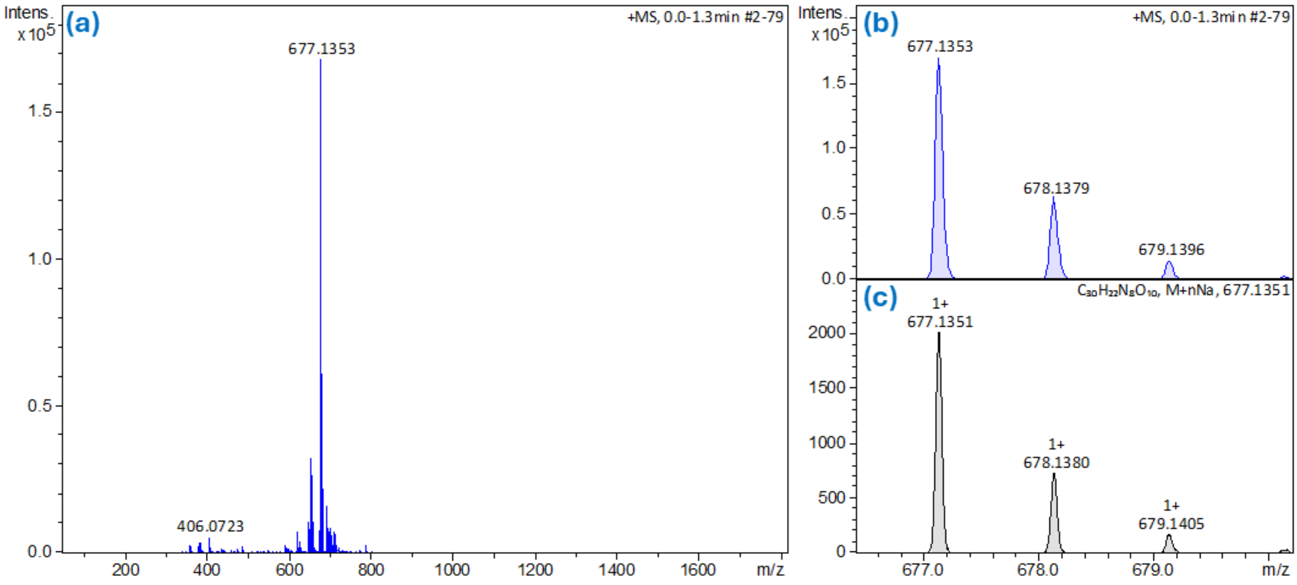

Figure S31. (a) HRMS spectrum (ESI-QToF) of compound **9a** (a), the expanded regium of the spectrum detailing the sodium-cationized molecule [M+Na]$^+$ and, (c) the simulated spectrum for $C_{30}H_{22}N_8O_{10}Na$ [M+Na]$^+$.

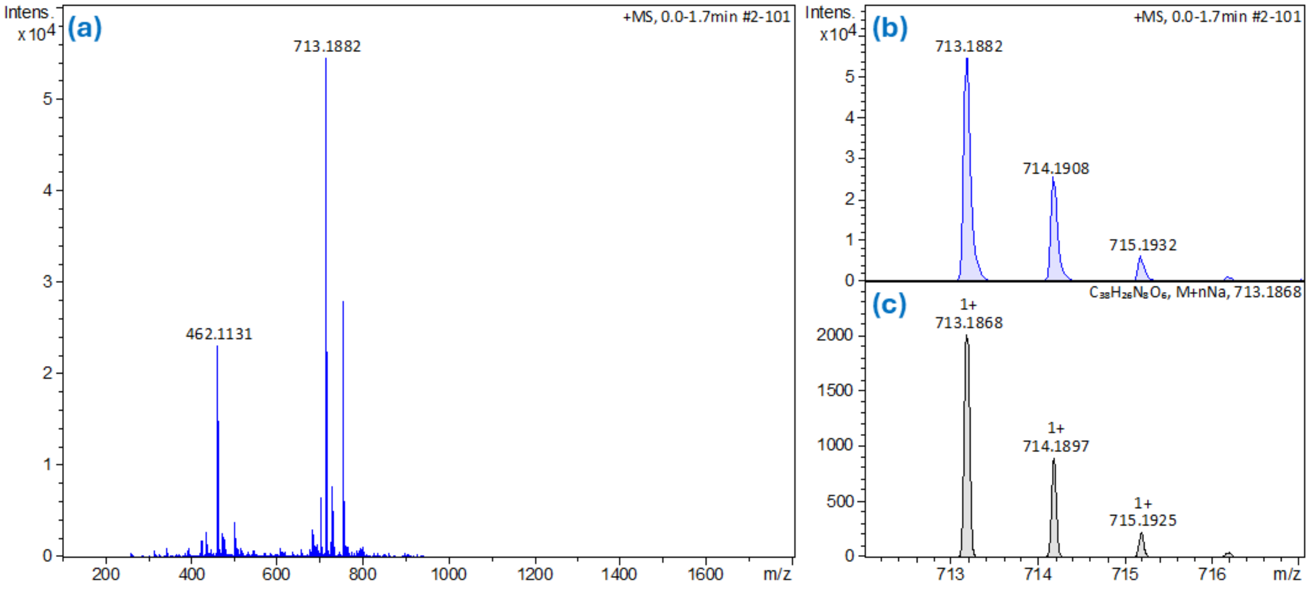

Figure S32. (a) HRMS spectrum (ESI-QToF) of compound **9b** (a), the expanded regium of the spectrum detailing the sodium-cationized molecule [M+Na]$^+$ and, (c) the simulated spectrum for $C_{38}H_{26}N_8O_6Na$ [M+Na]$^+$.

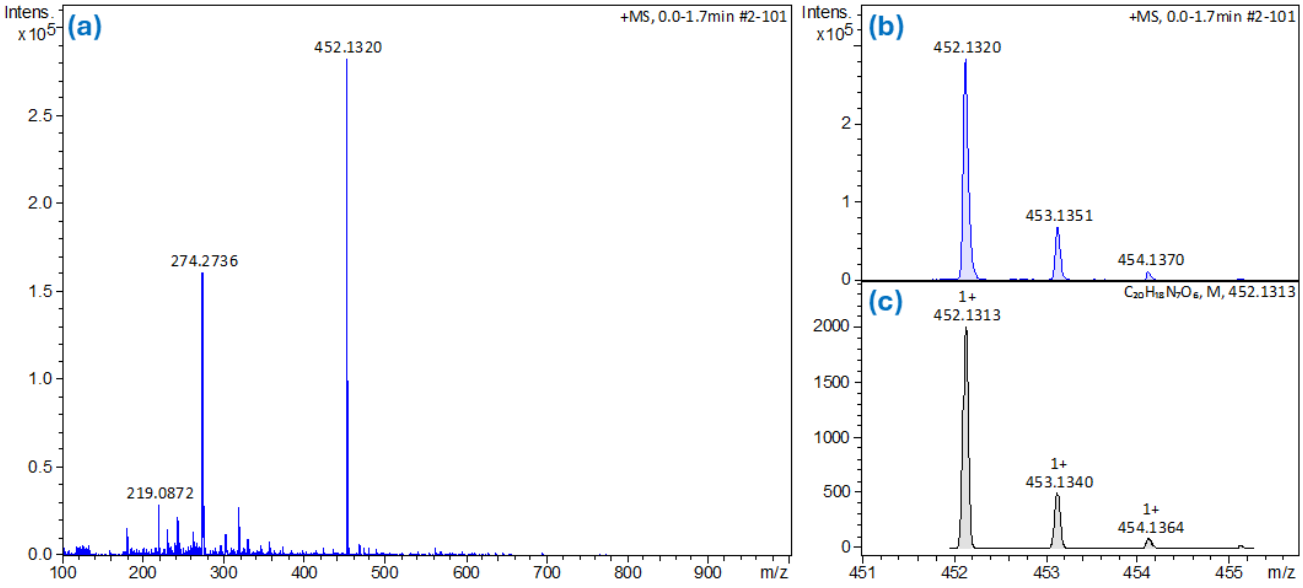

Figure S33. (a) HRMS spectrum (ESI-QToF) of compound **10a** (a), the expanded regium of the spectrum detailing the protonated molecule $[M+H]^+$ and, (c) the simulated spectrum for $C_{20}H_{18}N_7O_6$ $[M+H]^+$.

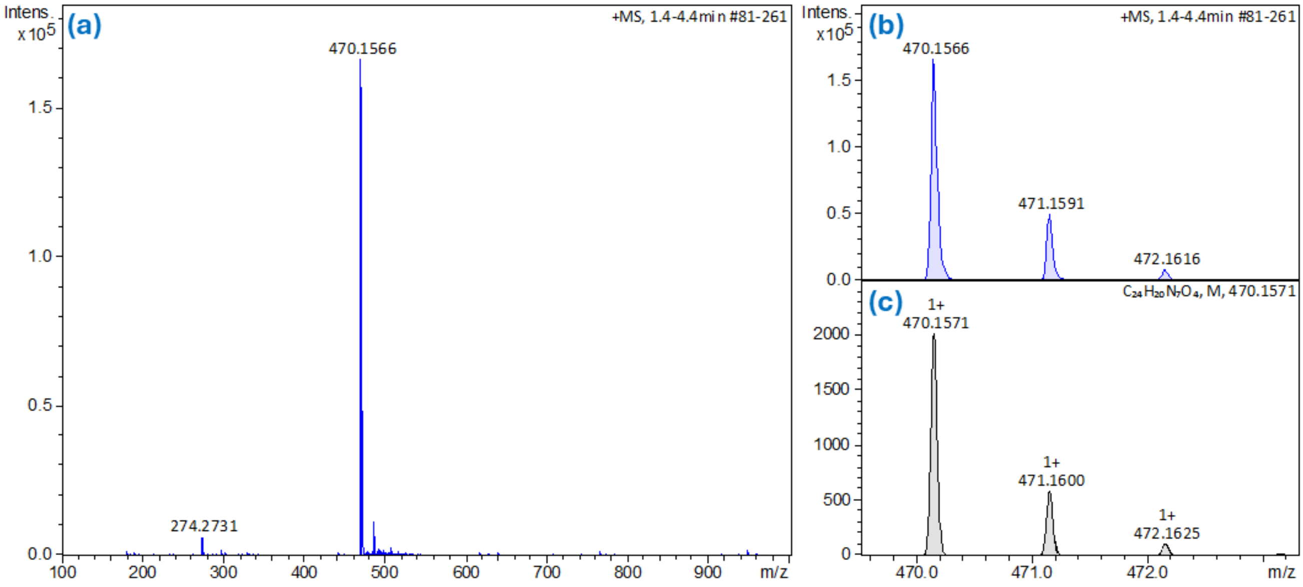

Figure S34. (a) HRMS spectrum (ESI-QToF) of compound **10a** (a), the expanded regium of the spectrum detailing the protonated molecule $[M+H]^+$ and, (c) the simulated spectrum for $C_{24}H_{20}N_7O_4$ $[M+H]^+$.
